# Perception of power quality disturbances using Fourier, Short-Time Fourier, continuous and discrete wavelet transforms

**DOI:** 10.1038/s41598-024-53792-9

**Published:** 2024-02-11

**Authors:** M. S. Priyadarshini, Mohit Bajaj, Lukas Prokop, Milkias Berhanu

**Affiliations:** 1 Department of Electrical and Electronics Engineering, K. S. R. M. College of Engineering (Autonomous), Kadapa, 516005 India; 2 Department of Electrical Engineering, Graphic Era (Deemed to Be University), Dehradun, 248002 India; 3 Hourani Center for Applied Scientific Research, Al-Ahliyya Amman University, Amman, Jordan; 4 Graphic Era Hill University, Dehradun, 248002 India; 5 Applied Science Research Center, Applied Science Private University, Amman, 11937 Jordan; 6 ENET Centre, VSB—Technical University of Ostrava, 708 00 Ostrava, Czech Republic; 7 Department of Electrical and Computer Engineering, College of Engineering, Addis Ababa Science and Technology University, Addis Ababa, Ethiopia

**Keywords:** Continuous wavelet transform, Discrete wavelet transform, Fourier transform, Short-time Fourier transform, Power quality disturbances, Energy science and technology, Engineering, Mathematics and computing

## Abstract

Electric power utilities must ensure a consistent and undisturbed supply of power, with the voltage levels adhering to specified ranges. Any deviation from these supply specifications can lead to malfunctions in equipment. Monitoring the quality of supplied power is crucial to minimize the impact of fluctuations in voltage. Variations in voltage or current from their ideal values are referred to as "power quality (PQ) disturbances," highlighting the need for vigilant monitoring and management. Signal processing methods are widely used for power system applications which include understanding of voltage disturbance signals and used for retrieval of signal information from the signals Different signal processing methods are used for extracting information about a signal. The method of Fourier analysis involves application of Fourier transform giving frequency information. The method of Short-Time Fourier analysis involves application of Short-Time Fourier transform (STFT) giving time–frequency information. The method of continuous wavelet analysis involves application of Continuous Wavelet transform (CWT) giving signal information in terms of scale and time where frequency is inversely related to scale. The method of discrete wavelet analysis involves application of Discrete Wavelet transform (DWT) giving signal information in terms of approximations and details where approximations and details are low and high frequency representation of original signal. In this paper, an attempt is made to perceive power quality disturbances in MATLAB using Fourier, Short-Time Fourier, Continuous Wavelet and Discrete Wavelet Transforms. Proper understanding of the signals can be possible by transforming the signals into different domains. An emphasis on application of signal processing techniques can be laid for power quality studies. The paper compares the results of each transform using MATLAB-based visualizations. The discussion covers the advantages and disadvantages of each technique, providing valuable insights into the interpretation of power quality disturbances. As the paper delves into the complexities of each method, it takes the reader on a journey of signal processing complexities, culminating in a nuanced understanding of power quality disturbances and their representations across various domains. The outcomes of this research, elucidated through energy values, 3D plots, and comparative analyses, contribute to a comprehensive understanding of power quality disturbances. The findings not only traverse theoretical domains but also find practical utility in real-world scenarios.

## Introduction

Continuous and reliable electric power is generated and supplied to various loads. The production of high-quality electric power adhering to specific standards is essential for optimal performance. Sankaran^1^ defines power quality in a comprehensive manner, encompassing the boundaries within which electrical systems should operate to achieve the desired performance. The IEEE Standard Dictionary of Electrical and Electronics characterizes power quality as the concept of powering and grounding sensitive equipment to ensure proper operation. Reference^2^ provides a comprehensive overview of power quality disturbances, categorizing them based on their characteristics. Various devices are employed to mitigate the impact of factors that can affect the quality of electric power.

Signals are transformed in order to get information from them. A processed signal is one that has undergone any of the numerous mathematical changes. Signal processing techniques based on various transformation methods can be used to analyze, diagnose, and identify power quality issues. In Fourier analysis a signal is decomposed into a sum of sinusoidal signals of different frequencies. Short-time Fourier Transform (STFT) has been used in power quality analysis as it can be applied to non-stationary signals. Analog voltage and current signals are transformed to sampled digital values for automatic power quality monitoring. Lieberman et al.^3^ reviewed that some transforms give time and frequency domain information and can be used to classify power quality disturbances.

Initiation of preventive action for improved power quality requires correct recognition of the event. Fourier transform (FT), short time Fourier transform (STFT) and wavelet transform (WT) are widely used for information extraction from PQ events. According to Collins et al.^4^, sampled data from disturbances is used to convert into different categories having certain attributes. “Phase shift and missing voltage” are examined and the measurement requirements of the instruments are also addressed in^4^. The fundamental voltage component is used by Wang et al.^5^ for situations resulting in magnitude change. Santoso et al.^6^ analyzed that dilation of single prototype function results in analysis and decompose a signal into different scales and levels of resolutions. An algorithm is implemented by Naidoo et al.^7^ for extracting non-stationary sinusoidal signal out of a given signal as input and used for estimation of amplitude, phase and frequency during voltage sag. Tanaboylu et al.^8^ stated that the difference between the transient and ideal sine waveforms is used for disturbance evaluation. A method is proposed by Sushama et al.^9^ having steps of de-noising, detection of duration and cause of power quality disturbances using discrete wavelet transform. Discrete wavelet transform respectively provides short and long windows for high and low frequency components. The transients are localized in the process of analysis.

Wavelet analysis-based techniques are used for detecting, localizing and classifying different transients. A new event detection scheme for power quality analysis based on the statistical analysis of adaptive decomposition signals is proposed. Based on WT and de-noising, the system proposed by Yang et al.^10^ is able to detect disturbances in noisy surroundings. Wavelet transform is used as part of a procedure for accurate detection and localization of sag. A. C. Parsons et al.^11^ proposed method for identifying the start and stop time of disturbances. Translation and scaling refer to generation of wavelets from a single basic wavelet called as mother wavelet. Low scale, high-frequency components are termed as ‘Details and high-scale, low-frequency components are termed as ‘approximations’ of a signal. Wavelet and scaling of functions are used as to decompose a signal at different resolutions and is termed as multiresolution analysis (MRA). Detailed and approximated versions are generated by wavelet and scaling functions. An approximation contains tendency of the original signal. Details are obtained through a succession of convolution process^12^. Energy of detail coefficients termed as “detail-spectrum-energy^13^” of the normalized phase currents is used for a scale value of one by Costa et al. to perform the functions of detection and classification. Time–frequency plane is used and measured characteristics and benchmark values are compared to detect disturbances in signals. Poisson et al.^14^ located transients in the width of the signal and measured duration and magnitude of sags.

According to Singh et al.^15^ complex wavelet transform suffers from certain limitations. It is concluded in^16^ that first level wavelet coefficient energies are suited to detect very short duration components. A new methodology is proposed by Costa et al.^17^ for voltage sag characterization using wavelet transform. WT coefficients (WTCs) of details determine disturbance occurrence. Wavelet analysis of PQ events depends on the chosen mother wavelet and is crucial for analysis. A signal can be estimated and transformed from time to time–frequency domain using WT. It is not possible to generalize mother wavelet and levels of decomposition due to different applications and conditions. Ibrahim et al.^18^ stated wavelets as powerful tool for PQ signal analysis.

A fundamental understanding of the many notations used to express power quality is required to extract significant information from signals using signal processing techniques. The definition of power quality in the Institute of Electrical and Electronics Engineers (IEEE) dictionary^19^ focuses on the "powering and grounding" components of devices. Only with this basic understanding can effective signal processing be used to analyze and improve power quality. Power produced must be interruption free or disturbance free. The International Electrotechnical Commission (IEC)^20^, defined power quality in terms of “characteristics of electricity evaluated against a set of reference parameters”. Wavelet basis functions along with their properties are explained in^21^ with an emphasis on choice of scales. Mallat^22^ explained about the suitability of wavelet transform for accurate signal description having “fully scalable window”.

A major obstacle to preserving the stability and reliability of electrical systems is power quality disturbances. For efficient mitigation and enhanced power system performance, it is essential to identify and characterize these disturbances. In order to improve our comprehension of power quality disruptions, this research tackles the need for sophisticated signal processing techniques. It seeks to solve shortcomings in current approaches that could lead to the loss of subtle patterns and transitions in the signals.

By methodically applying and contrasting four well-known signal processing techniques—the Fourier transform, the Discrete Wavelet transform (DWT), the Continuous Wavelet transform (CWT), and the Short-Time Fourier transform (STFT)—to power quality disturbances, this research adds to the body of knowledge already in existence. The study explores the subtleties of each technique and assesses how well it reveals particular features of the disturbances.

The newly developed use of DWT for signal decomposition into multiresolution components stands out as a noteworthy contribution. The study not only finds low and high-frequency representations but also excels at catching transitions and abrupt shifts within the signals by offering a deep examination of approximations and details. This unique method overcomes the limitations of previous methods by providing a more sophisticated knowledge of power quality disturbances and their representations across domains. The results of this study provide a refined and thorough approach for power quality assessment, which adds significant value to the field of power system analysis. In order to promote improvements in power system stability and reliability, future research will be guided by the comparative analysis provided in this work when choosing suitable signal processing algorithms based on particular characteristics of power quality disturbances.

## Power quality disturbance signals

Electric power utilities provide voltage that often experiences undesirable variations such as transients, sags, swells, interruptions, voltage imbalances, DC offsets, harmonics, noise, and fluctuations. Ensuring a constant and stable voltage supply is crucial for maintaining the quality of power, and all these variations fall within the overarching category of power quality disturbances. The analysis of disturbance signals plays a vital role in identifying and implementing appropriate preventive measures.

To analyze various voltage variations and proactively address abrupt changes in the connected load, signal processing techniques prove instrumental. The detection of voltage signal variations is crucial for implementing effective preventive measures. Transforming signals, which are temporal functions, into the time and frequency domain facilitates a more insightful interpretation of the original signal in the time domain. The signals under consideration encompass sag, swell, interruption, transient, harmonics, fluctuations, and flicker, alongside a sinusoidal signal utilized as a reference. Each of these signals manifests a discernible deviation, either in magnitude or frequency, from the pristine sinusoidal form of voltage over specific durations. The paper delineates the definitions of power quality disturbance signals and elucidates the application of Fourier transform, short-time Fourier transform, continuous wavelet transform, and discrete wavelet transforms to power quality disturbances. The visual identification of disturbance signals through diverse transforms in MATLAB streamlines the categorization of disturbances, thereby enhancing power quality evaluation.

### Generation of power quality disturbance signals

Mathematical modelling is carried out by parametric equations and the equations used for developing MATLAB code for different disturbance signal generation are presented with the description of various parameters governing the equations. In order to apply signal processing methods, the basic step is to generate the signals. Due to changes in voltage in terms of any or all of magnitude, duration and frequency, there will be a deviation from pure sinusoidal form. Certain parameters define signals. Disturbances create signals, which are defined by waveforms with a fundamental frequency of 50Hz and a voltage magnitude of 1 per unit (pu) lasting 0.25 s. The term "pu" refers to a dimensionless number that represents measurements per unit.

Modeling power quality disturbances is critical in assessing power quality. Analyzing voltage disturbance waveforms leads to the discovery of power quality events. In^23^, a framework based on numerical models is used to generate various power quality waveforms. The term $A$ represents the maximum value of the supply voltage $V\left(t\right).$ Equation (1) represents pure sinusoidal supply voltage without any distortions with amplitude $A$ and is given as:

1 $$V\left(t\right)=Asin(\omega t)$$

In all the equations defined for transient, interruption, sag and swell the terms, $u\left({t}_{1}\right),u\left({t}_{2}\right), u\left(t-{t}_{1}\right)$ and $u\left(t-{t}_{2}\right)$ represent amplitude of unit step functions defined for period’s ${t}_{1}, {t}_{2},$ duration $\left(t-{t}_{1}\right)$ and duration $(t-{t}_{2})$ respectively. For voltage interruption, sag and swell the duration $({t}_{2}-{t}_{1})$ is between $T$ and $9T$, where $T$ represents time period of the sinusoidal voltage signal. Values of ${t}_{1}$ and ${t}_{2}$ are 0.08 and 0.16 s and ${t}_{2}$ is greater than ${t}_{1}$. The equations dictating each power quality disturbance are contingent on controlled parameters.

Choice of values of various parameters taken in literature depends on the necessity that generated signals must depict the actual conditions in a controlled manner and definitions given by IEEE must not be deviated. It is very important to choose different parameter values in such a manner that the waveforms are according to their standard definitions. Different simulation tools used for power system analysis are mentioned in^24^.

#### Transient

The term transient refers to an undesirable and short event. It can be a unidirectional impulse of positive or negative polarity. It can also be an oscillatory wave with damping and first peak occurring in either polarity^2^. Transients are mainly due to lightning strikes on transmission lines resulting in dangerously high potential differences.

Oscillatory transients are numerically modelled^15^ by Eq. (2) as:

2 $$V\left( t \right) = A\left[ {{\text{sin}}\,\omega t + \alpha e^{{\frac{{ - (t - t_{1} )}}{\tau }}} {\text{sin}}\,\omega _{n} (t - t_{1} )(u\left( {t_{2} } \right) - u\left( {t_{1} } \right))} \right]$$

Angular frequencies of supply voltage and transients are $\omega =2\pi f$ and ${\omega }_{n}=2\pi {f}_{n}$. The terms α, τ and ${f}_{n}$ represent magnitude, settling time and oscillatory frequency respectively for the transient. In the equations for transients, the ranges taken for α, τ and ${f}_{n}$ are 0.1 to 0.8, 0.008 to 0.04 s and 300 to 900 Hz respectively. The transient disturbance is defined for period ${t}_{1}$ as:

3 $$V\left(t\right)={\text{sin}}\left(\mathrm{\omega t}\right)+{\mathrm{\alpha }}^{-\left({\text{t}}-{{\text{t}}}_{1}\right)/\uptau }{\text{sin}}{\upomega }_{{\text{n}}}\left({\text{t}}-{{\text{t}}}_{1}\right)$$

**Figure 1:**
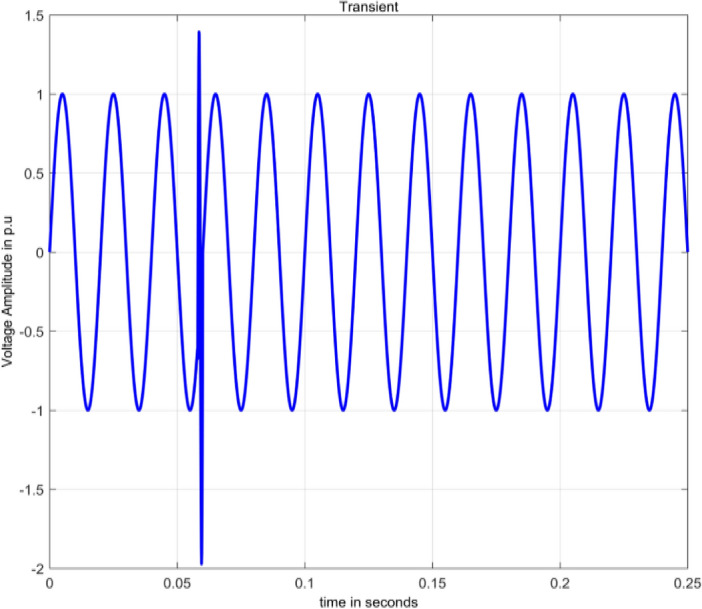
Transient.

Figure 1 depicts transient, simulated in MATLAB using Eq. (3). The magnitude of voltage varies from 1.381 to  − 1.972 pu for a very short duration.

#### Interruption

An interruption is identified by the loss of supply voltage or load current. Specifically, it happens when the supply voltage or load current drops to less than 0.1 per unit (pu) and lasts for no more than 1 min^2^. Interruption is numerically modelled as in^15^ in Eq. (4).

4 $$V(t)=A\left(1-\alpha \left(u\left(t-{t}_{1}\right)-\alpha \left(u\left(t-{t}_{2}\right)\right)\right)\right){\text{sin}}\,\omega t$$

**Figure 2:**
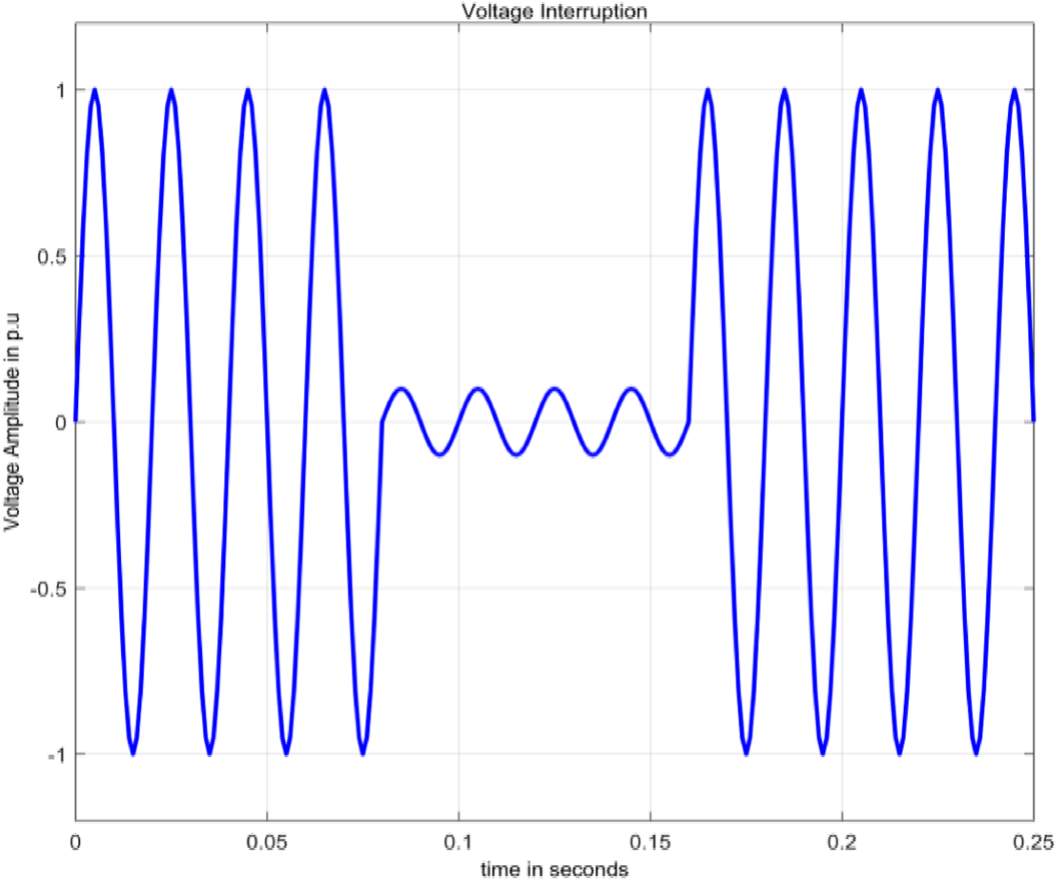
Interruption.

Using MATLAB to simulate Eq. (4), Fig. 2 displays a waveform with an interruption, illustrating a complete loss of voltage for a specific duration. The range for the parameter $\alpha$ is 0.9 to 1.

#### Voltage sag

Sag is decrease in rms voltage from 0.1 pu and 0.9 pu for duration of 0.5 cycles to 1 min^2^. Voltage sag is numerically modelled as in^15^ is given by Eq. (5).

5 $$V\left(t\right)=A\left(1-\alpha \left(u\left(t-{t}_{1}\right)-\alpha \left(u\left(t-{t}_{2}\right)\right)\right)\right){\text{sin}}\,\omega t$$

**Figure 3:**
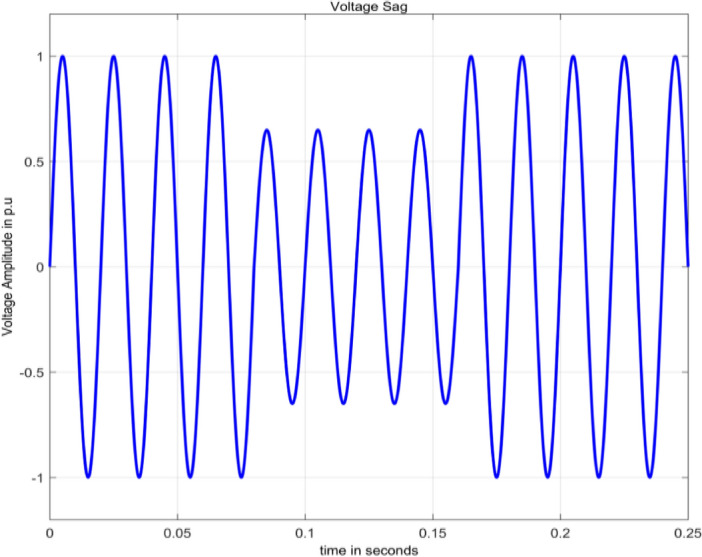
Voltage sag.

Figure 3 depicts a voltage sag, obtained through the simulation of Eq. (5) in MATLAB.

 The range for the parameter $\alpha$ is 0.1 to 0.9.

#### Voltage swell

Swell is characterized by an increase in root mean square (rms) voltage beyond 1.1 per unit (pu) up to 1.8 pu, lasting from 0.5 cycles to 1 min^2^. Voltage swell is numerically modelled as in^15^ is given by Eq. (6).

6 $$V\left(t\right)=A\left(1+\alpha \left(u\left(t-{t}_{1}\right)-\alpha \left(u\left(t-{t}_{2}\right)\right)\right)\right){\text{sin}}\,\omega t$$

**Figure 4:**
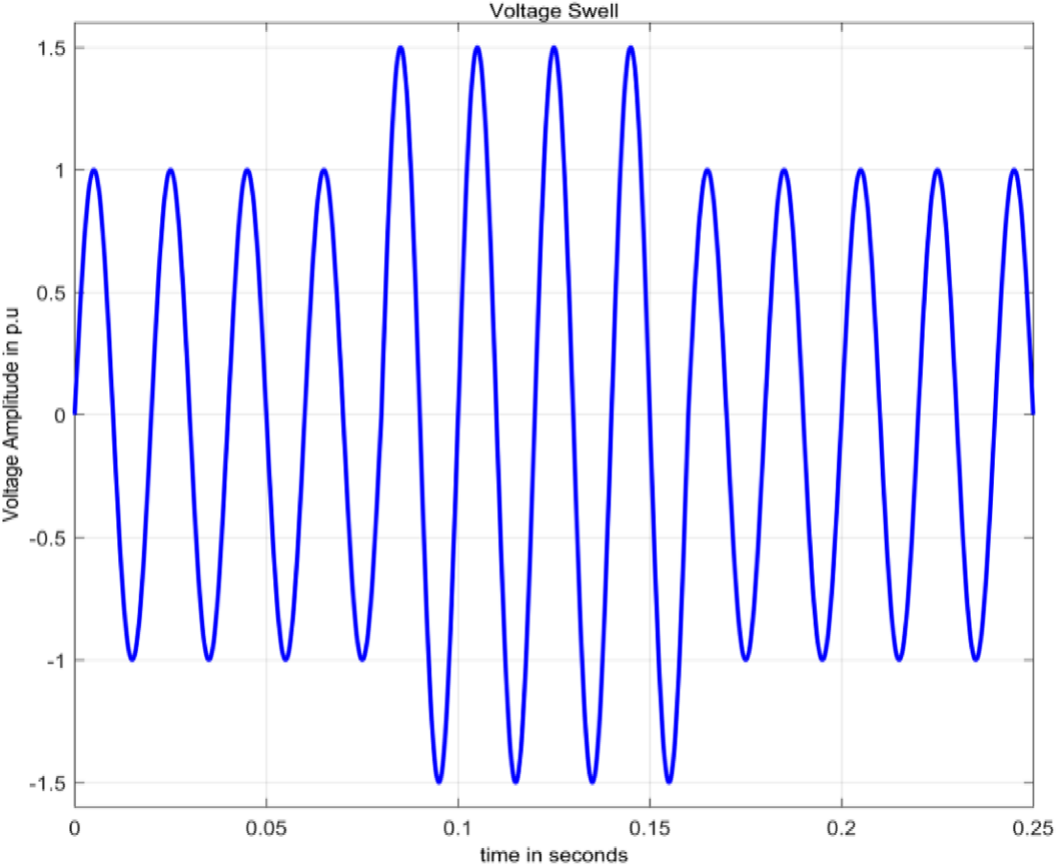
Voltage swell.

Figure 4 illustrates a voltage swell, achieved through the simulation of Eq. (6) in MATLAB. This graph signifies a sudden and temporary rise in voltage for a specific duration.

 The range for the parameter $\alpha$ is 0.1 to 0.9.

#### Harmonics

Harmonics fall within the realm of waveform distortion, representing voltages or currents with integer multiples of the fundamental frequency^2^. Harmonics are produced by loads having nonlinear characteristics and are numerically modelled as in^15^ and given by Eq. (7).

7 $$V\left(t\right)=A\sum {\alpha }_{n}{\text{sin}}\,(n\omega t),1\le n \&\sum_{i=1}^{n}{\alpha }_{i}^{2}=1$$

The magnitude of nth order harmonic is ${\alpha }_{n}$ and is summation of amplitudes of harmonic components.

8 $$V\left(t\right)={\alpha }_{1}{\text{sin}}\,\omega t+{\alpha }_{3}{\text{sin}}\,3\omega t+{\alpha }_{5}{\text{sin}}\,5\omega t+{\alpha }_{7}{\text{sin}}\,7\omega t$$

**Figure 5:**
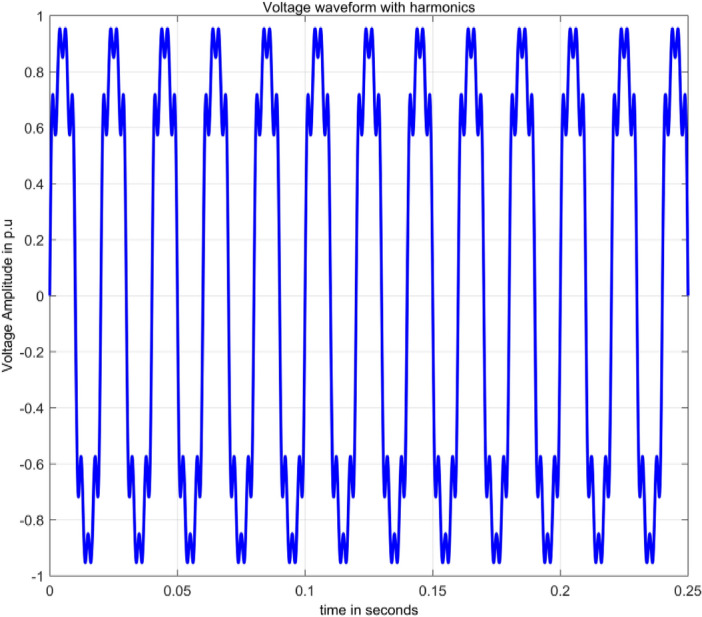
Harmonics.

By simulating Eq. (8) for harmonics, harmonics signal is obtained as depicted in Fig. 5.

#### Fluctuations

Fluctuations are systematic variations of envelope of voltage. There will be random changes and magnitude of voltage does not exceed 0.95 pu to 1.05 pu^2^.

9 $$V\left(t\right)=A(1+a{\text{sin}}\,(b\omega t))sin\,(\omega t)$$

**Figure 6:**
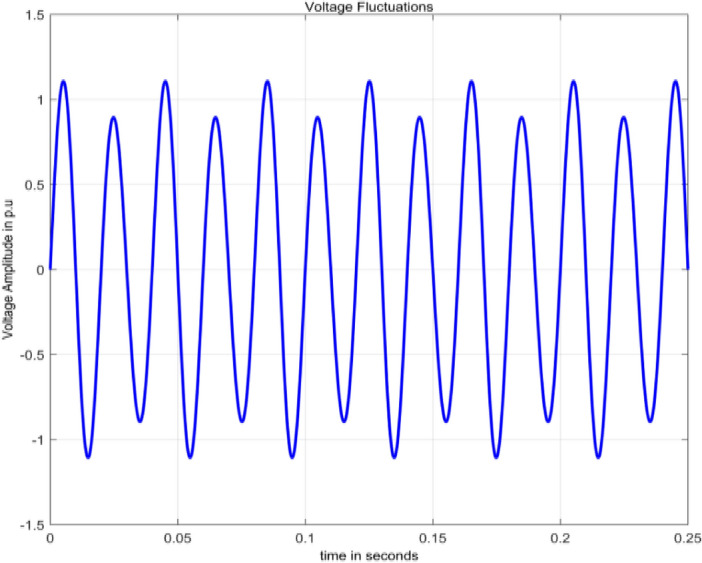
Voltage fluctuations.

Terms $a$ and $b$ are controlling parameters representing magnitude and integer multiple of frequency with ranges given as $0.1\le a\le 0.2$ and $0.4\le b\le 0.6$ . Waveform of fluctuations is shown in Fig. 6, obtained in MATLAB by simulating Eq. (9).

#### Flicker

Flicker is the consequence of voltage fluctuations affecting lighting intensity^2^. The voltage signal, as expressed in terms of flicker^25^, is defined by Eq. (10).

10 $$V\left(t\right)=\left[{A}_{1}+\sum_{i=1}^{I}{A}_{\mathit{fi}}{\text{sin}}\,\left({\omega }_{fi}t+{\Phi }_{fi}\right)\right]{\text{sin}}\,\left({\omega }_{1}t+{\Phi }_{1}\right)$$

${A}_{1}$, ${\omega }_{1}$ and ${\Phi }_{1}$ correspond to amplitude, angular frequency and phase angle of fundamental component of voltage. ${A}_{fi}$,  ${\omega }_{fi}$ and ${\Phi }_{fi}$ correspond to amplitude, angular frequency and phase angle of flicker component of voltage. The term $I$ refers to number of components of flicker.

**Figure 7:**
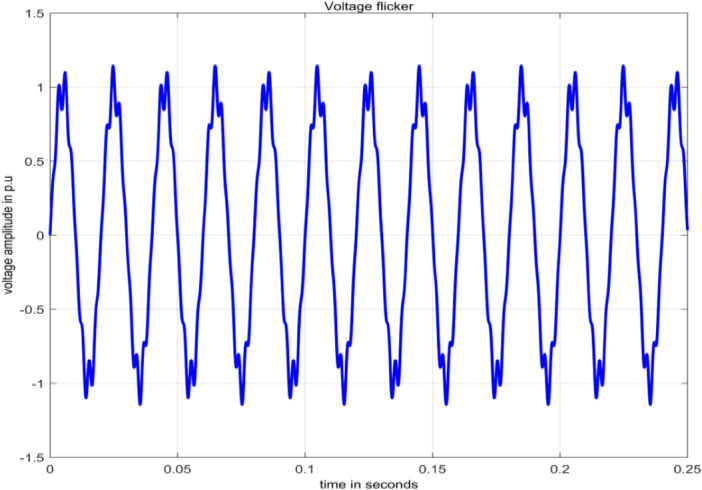
Voltage flicker.

In^25^, general procedure to get severity of flicker level is described in terms of several blocks involving extraction of fundamental signal, voltage envelope from a power signal fed as input and with an output of “instantaneous flicker level”. This process involves spectral analysis to identify flicker components. Flicker waveform is shown in Fig. 7 and is generated in MATLAB.

The generated power quality disturbances contain information about disturbance in terms of magnitude and duration. Preventive measures are to be taken for avoiding the disturbances.

## Application of Fourier transform

**Figure 8:**
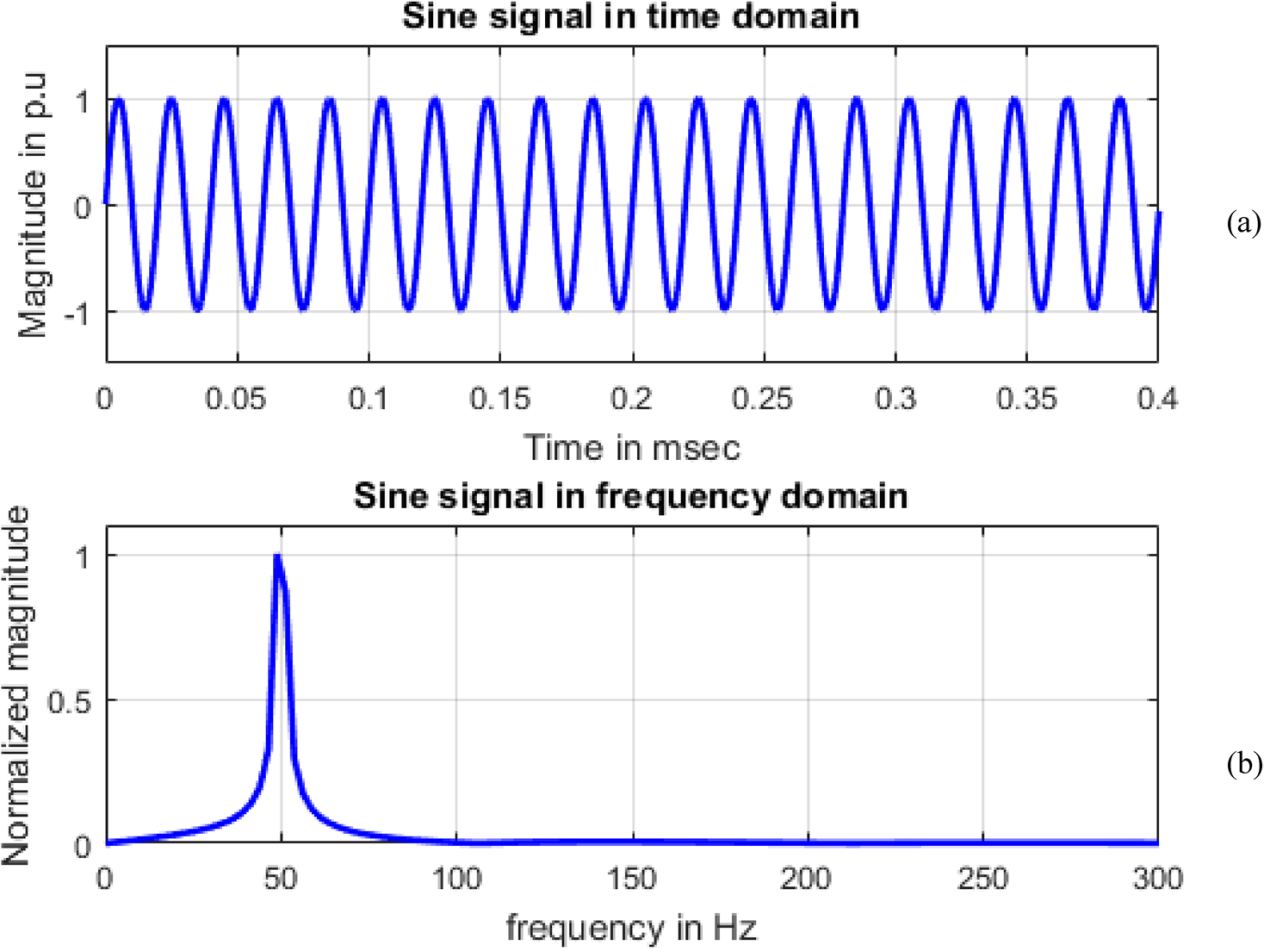
Sine signal (**a**) Time domain (**b**) Frequency domain.

**Figure 9:**
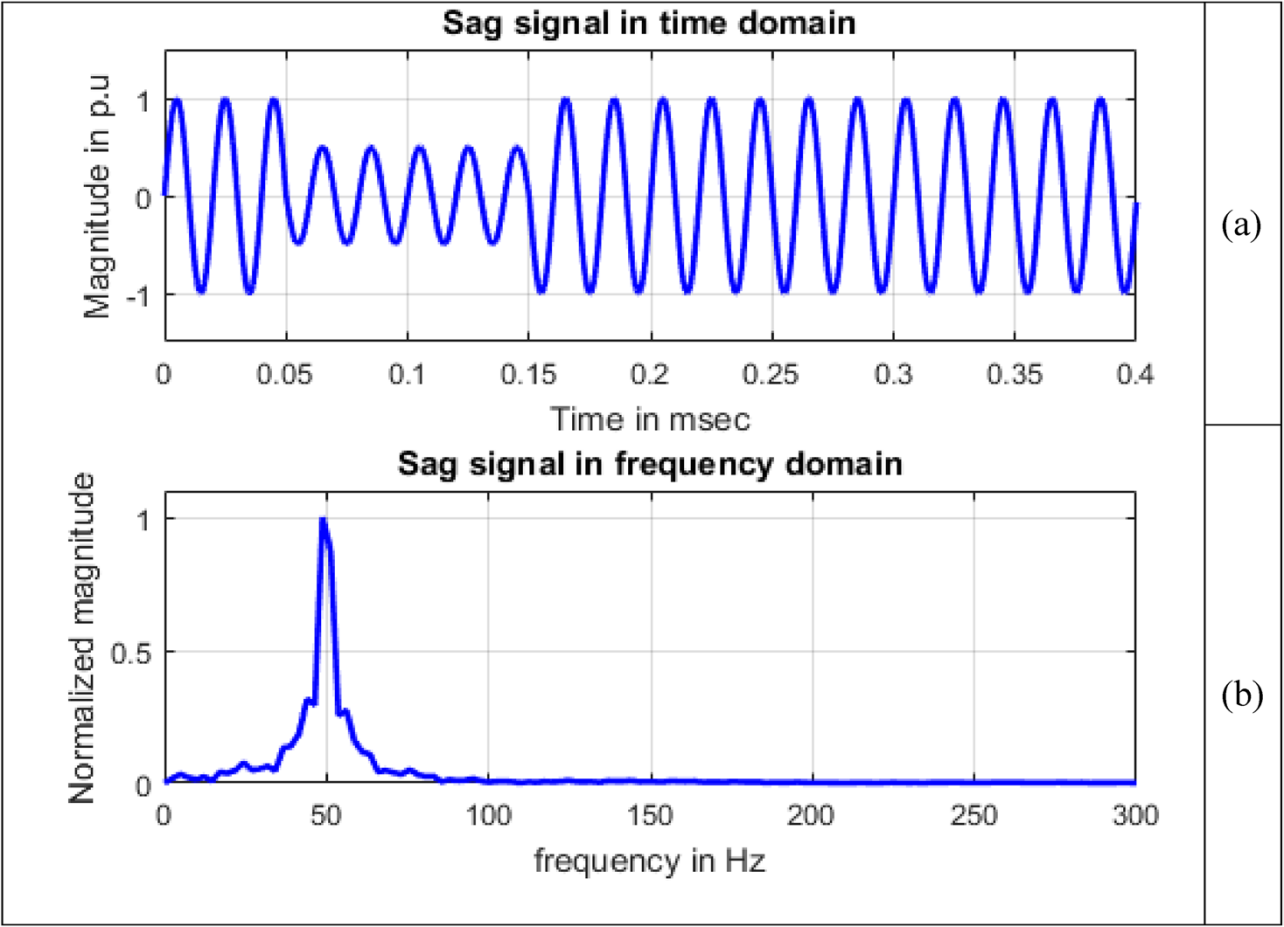
Sag signal (**a**) Time domain (**b**) Frequency domain.

**Figure 10:**
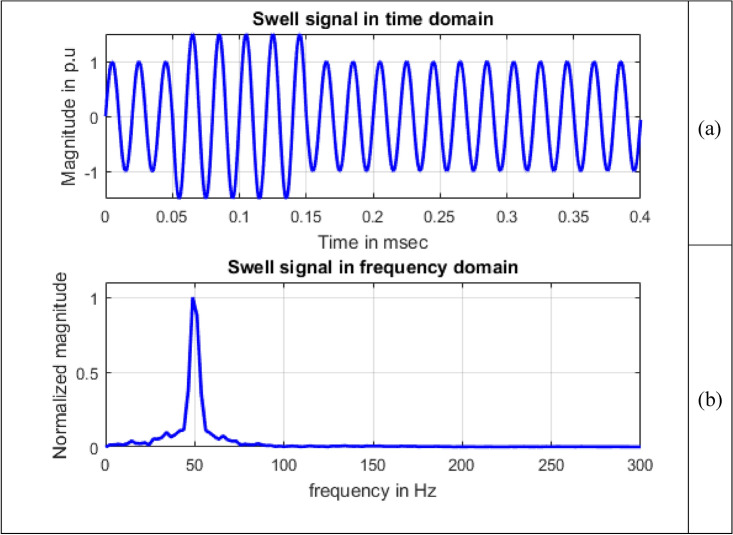
Swell signal (**a**) Time domain (**b**) Frequency domain.

**Figure 11:**
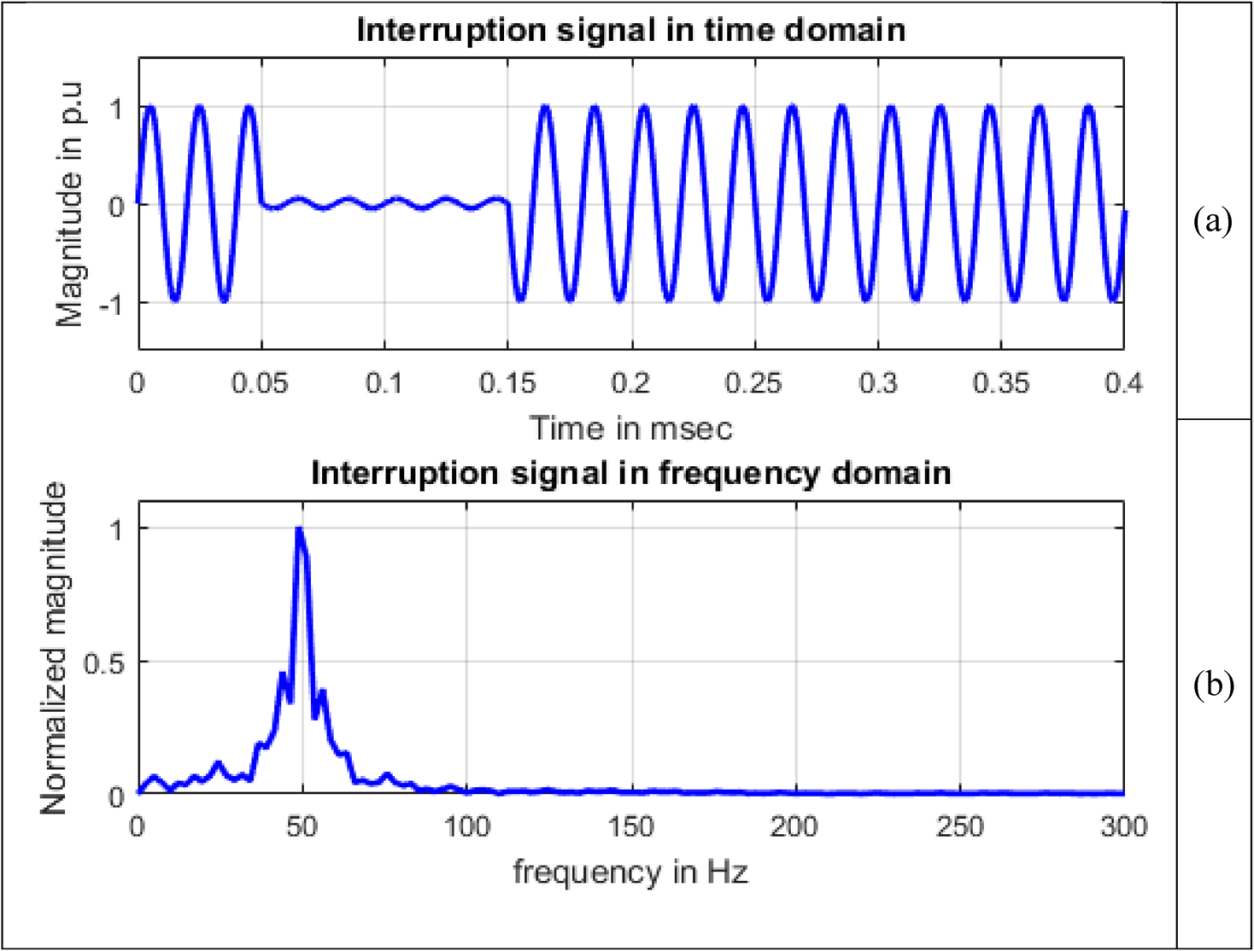
Interruption signal (**a**) Time domain (**b**) Frequency domain.

**Figure 12:**
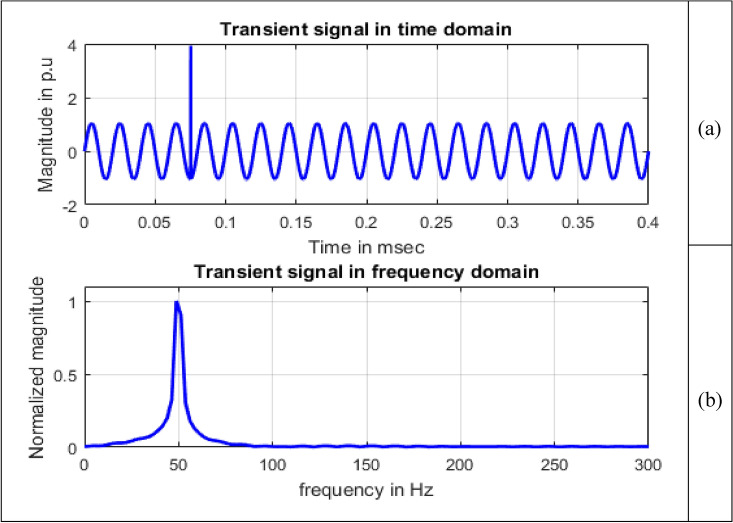
Transient signal (**a**) Time domain (**b**) Frequency domain.

Frequency information of the signals can be obtained by using Fourier transform. Maximum value of normalized magnitude is always unity. Length of the signal has 4001 samples. The spikes are termed as spectral components. To speed up calculations, as the signal length is not an exact power of 2, length of signal is taken to have 4096 samples and for single sided amplitude spectrum, 2048 samples are considered. For all the signals sine, sag, swell, interruption and transient shown in Figs. 8, 9, 10, 11 and 12, maximum value occur at a frequency of 50 Hz as all the signals contain only this frequency component. This frequency corresponds to sample number 21 out of considered 2048 frequency values. The mathematical equation given by Eq. (11) shows the transformation of signal $x(t)$ in time domain to $X(\omega )$ in frequency domain.

11 $$X\left(j\omega \right)={\int }_{-\infty }^{\infty }x(t){e}^{-j\omega t}dt$$

Single sided magnitude spectrum of PQ disturbance signals are depicted in Figs. 8, 9, 10, 11, 12, 13, 14 and 15.

**Figure 13:**
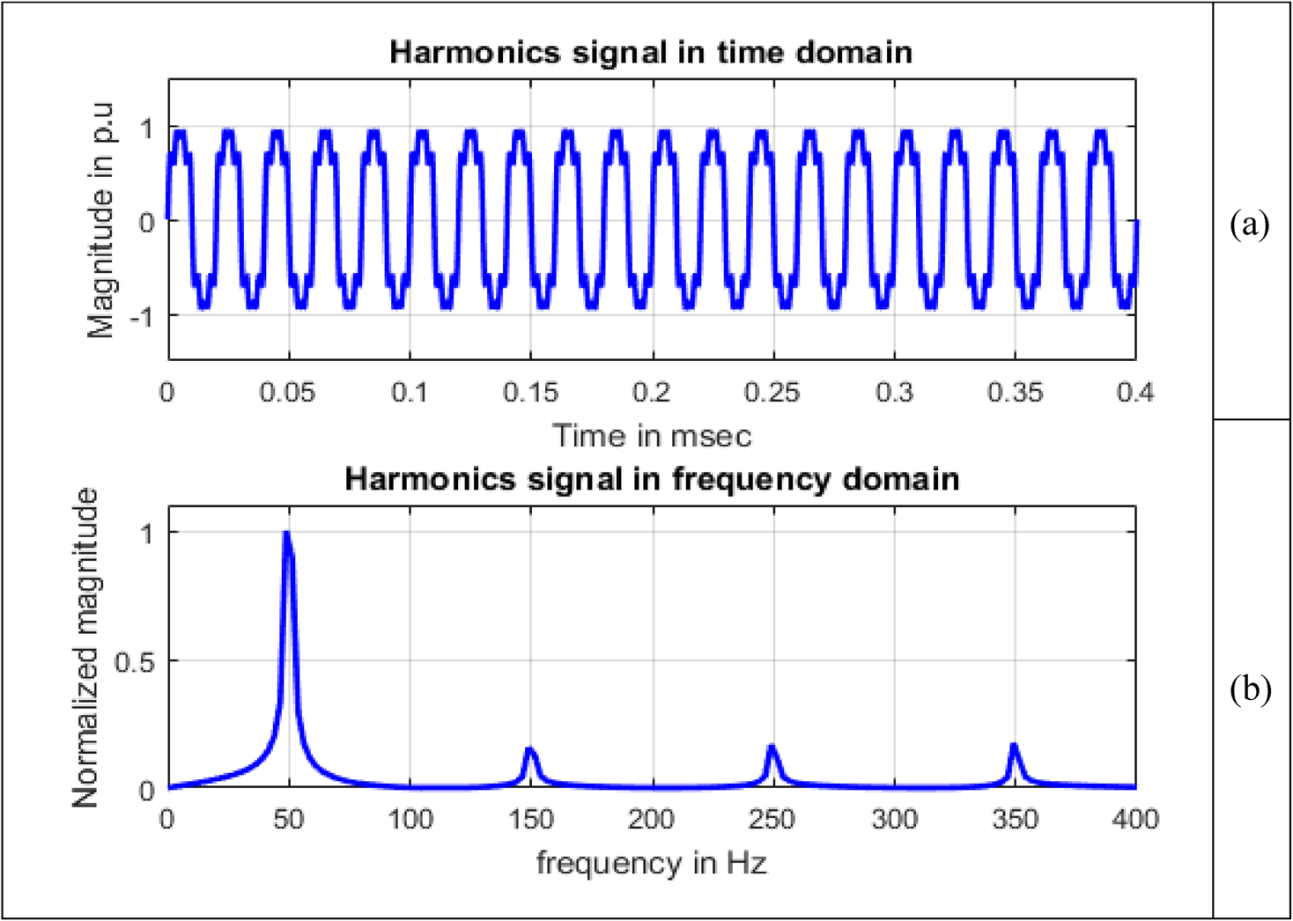
Harmonics signal (**a**) Time domain (**b**) Frequency domain.

**Figure 14:**
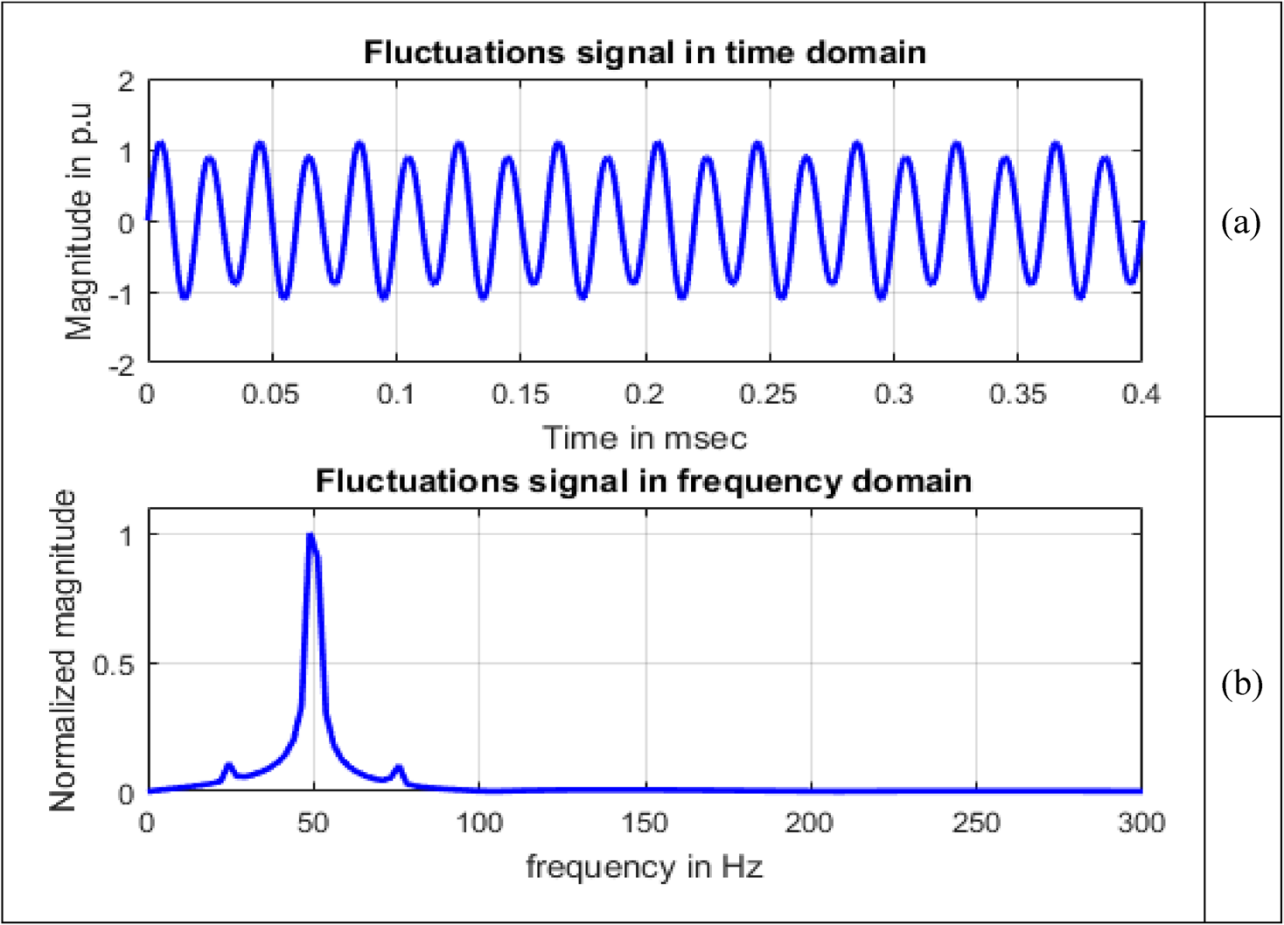
Fluctuations signal (**a**) Time domain (**b**) Frequency domain.

**Figure 15:**
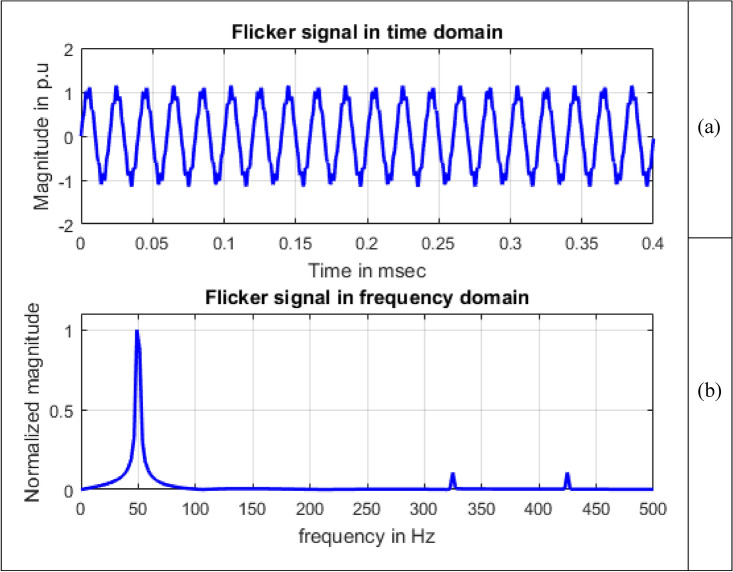
Flicker signal (**a**) Time domain (**b**) Frequency domain.

In Fig. 11, a variation in magnitude of 0.4575, 1 and 0.3925 is observed for frequencies 43.9453 Hz, 50 Hz and 56.1523 Hz.

In Fig. 13, other than the maximum value corresponding to frequency value 50 Hz, spikes are present at frequencies 150 Hz, 250 Hz and 350 Hz with sample numbers of 62, 103 and 144. This gives information that harmonics signal comprises of four signals, each at different frequency nearly equal to 50 Hz, 150 Hz, 250 Hz and 350 Hz. Normalized magnitude values at sample numbers of 21, 62, 103 and 144 are 1, 0.1559, 0.1666 and 0.1741.

In Fig. 14, other than the maximum value corresponding to frequency value 50 Hz, small spikes are present at frequencies 24.4141 Hz and 75.6836 Hz with sample numbers of 11 and 32 having magnitude 145.6590 and 135.3896 respectively with normalized magnitude values of 0.1065 and 0.0990. The maximum value of frequency considered on x-axis scale is 300 Hz as the magnitude values starting from 288.0859 Hz at sample 119 all the magnitude values which are either multiples of ten to the power of  − 4 or  − 5 up to last frequency 4997.6 Hz at sample 2048.

In Fig. 15, other than the maximum value corresponding to frequency value 50 Hz, small spikes are present at frequencies 324.7070 Hz and 424.8047 Hz with sample numbers of 134 and 175 having normalized magnitude 0.1072 and 0.1077 respectively. Magnitude values at sample numbers of 134 and 175 are 149.2614 and 149.9987. The maximum value of frequency considered on x-axis scale is 500 Hz as at 485.8398 Hz of sample 200 having normalized magnitude value 9.7066*10^−4^ has all decreasing magnitude values up to last frequency 4997.6 Hz at sample 2048.

It can be concluded that Fourier transform is effective for the signals whose frequency content is same at every point of time and determine existing frequency. But no adequate information is obtained related to sudden changes in voltage which are prone in practical point of view. The changes occurring in the signal are not localized in time i.e. the time at which these frequency components exist cannot be determined using Fourier transform. Information about frequency is absent in time domain and information about time is absent in frequency domain.

## Application of short time Fourier transform

Short time Fourier transform (STFT) is used to represent signals in time and frequency domains and depends on window size. In MATLAB, a function “*spectrogram*” is used to detect frequencies and their exact order to analyze signals using STFT. Color bars shown in figures are indicating low to high power levels of the signal. Spectrogram can be termed as visual representation of a signal as it varies with time. STFT plots represent frequency variations as a function of time with representing power at any instant by a color. The mathematical equation of STFT is given by Eq. (12).

12 $$STFT\,X\left( {\tau ,\omega } \right) = \mathop {\mathop \smallint \limits^{\infty } }\limits_{{ - \infty }} x\left( t \right)\omega (t - \tau )e^{{ - j\omega t}} dt$$

**Figure 16:**
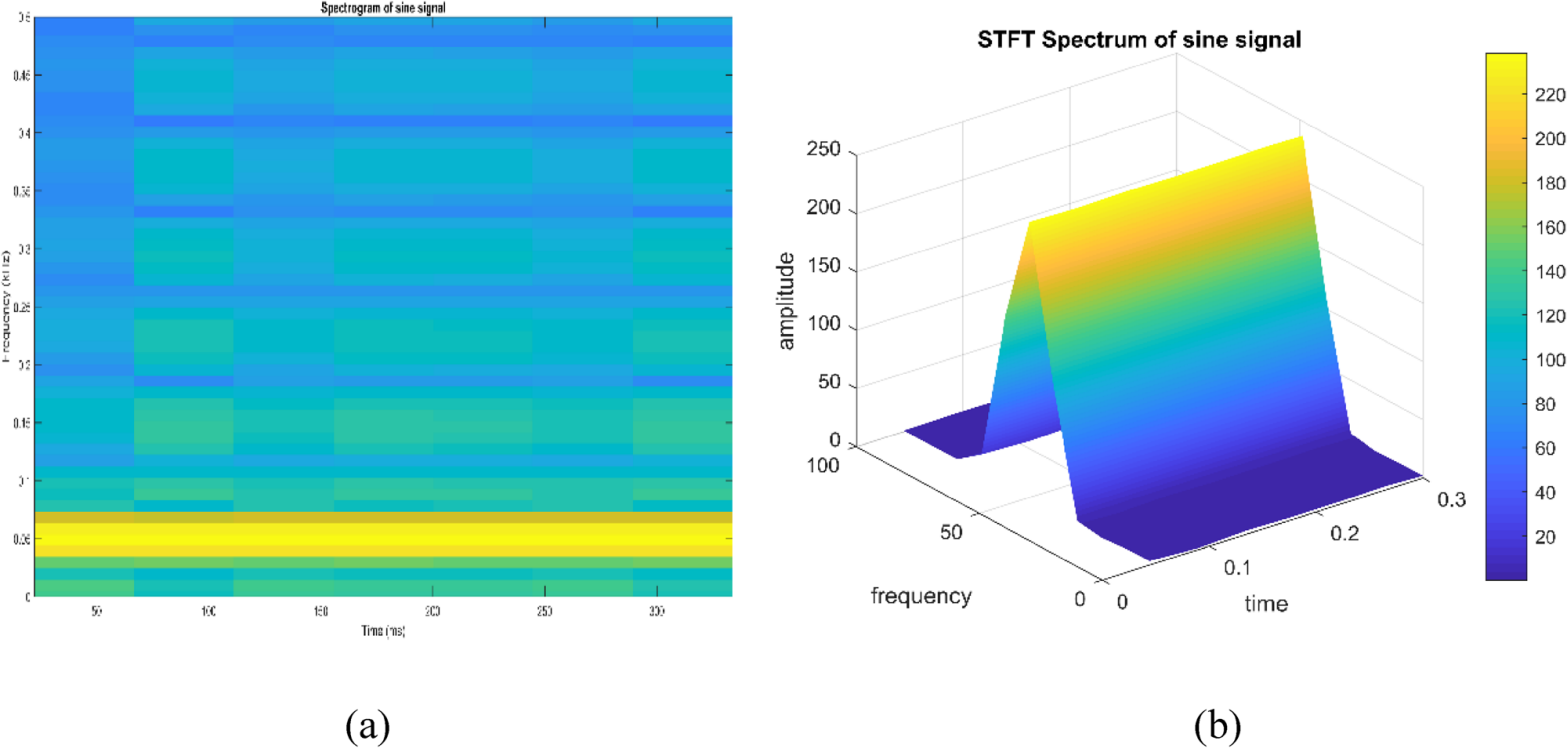
(**a**) Spectrogram of sine signal (**b**) STFT spectrum.

**Figure 17:**
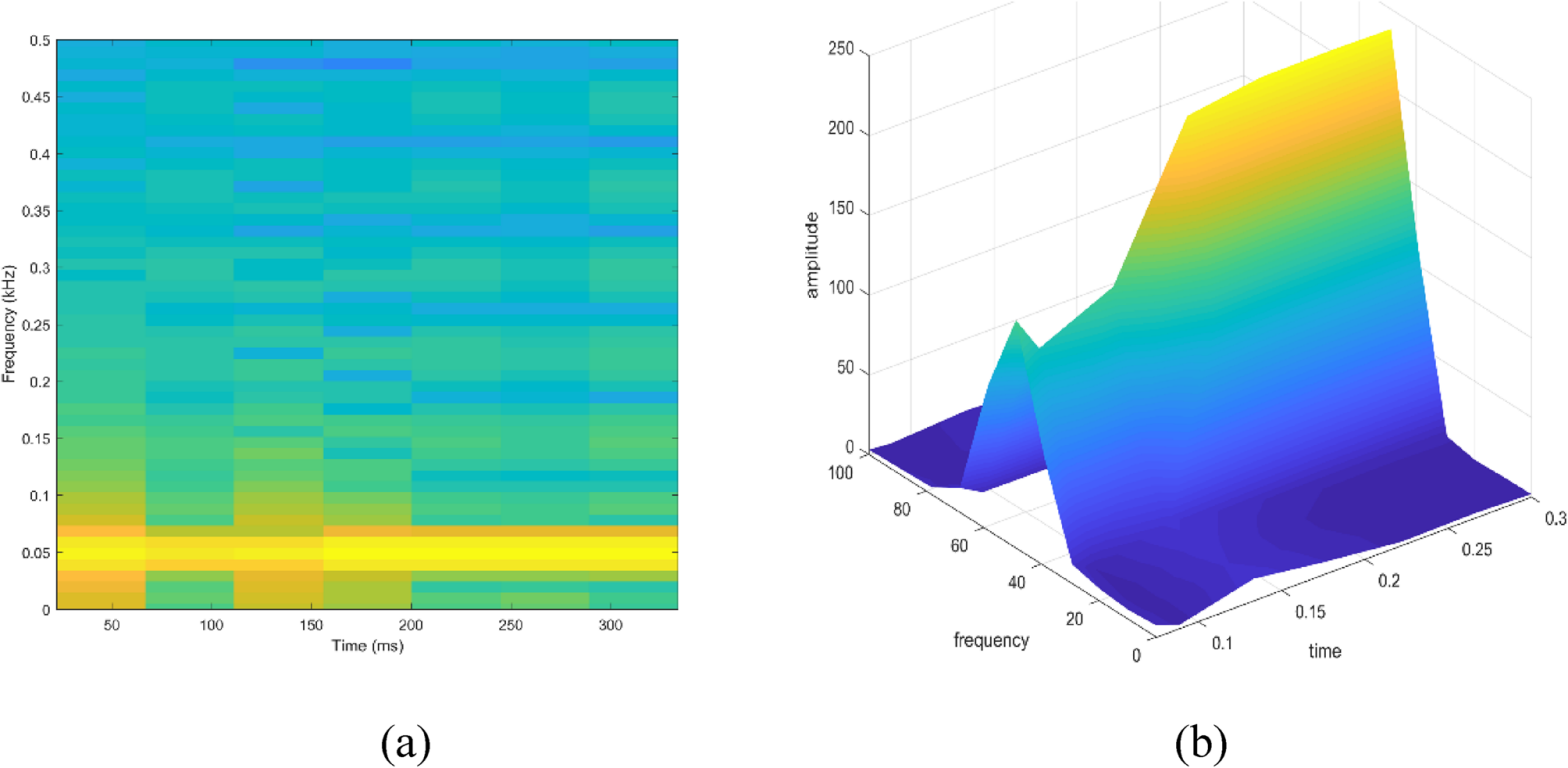
(**a**) Spectrogram of sag signal (**b**) STFT spectrum.

**Figure 18:**
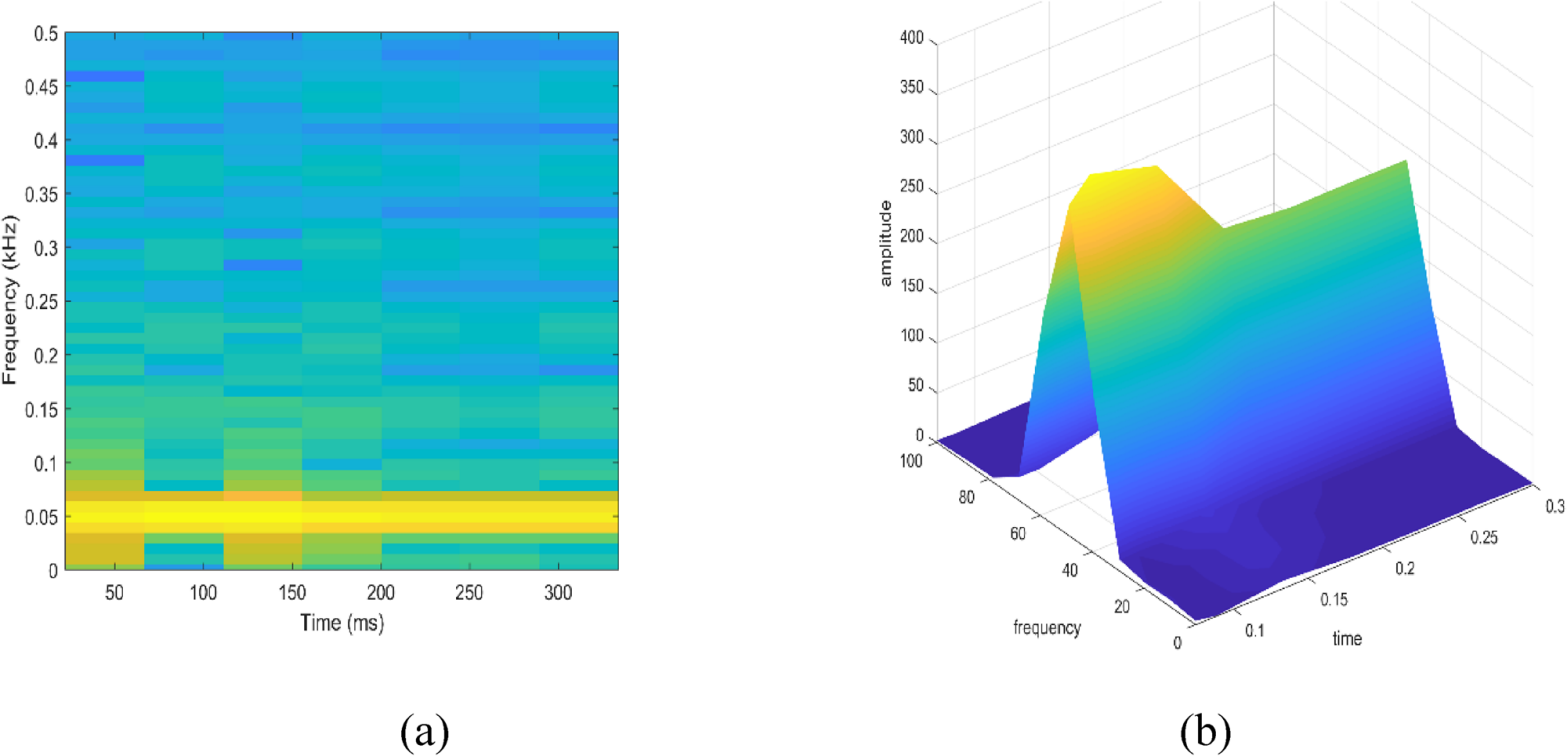
(**a**) Spectrogram of swell signal (**b**) STFT spectrum.

**Figure 19:**
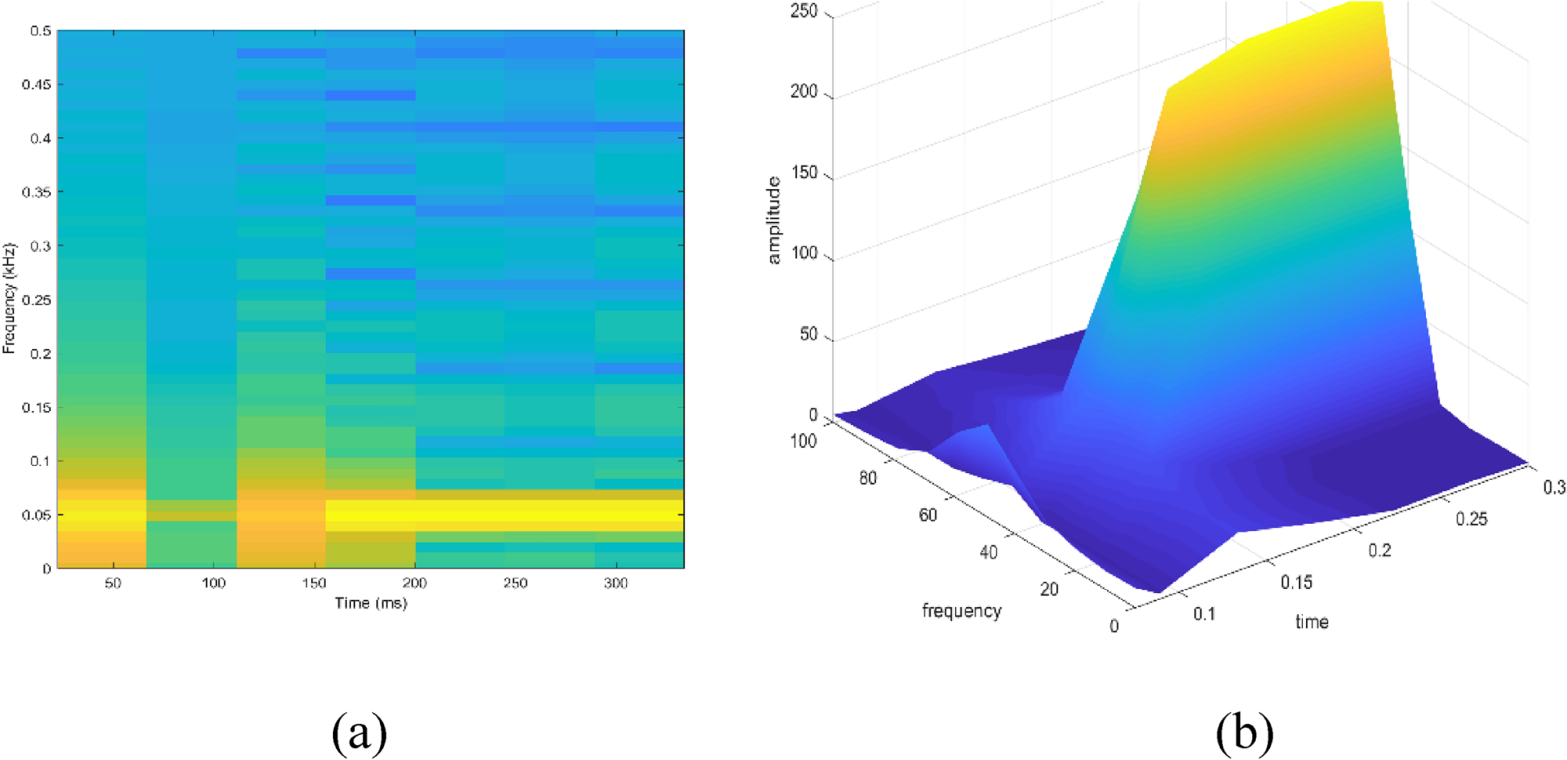
(**a**) Spectrogram of interruption signal (**b**) STFT spectrum.

**Figure 20:**
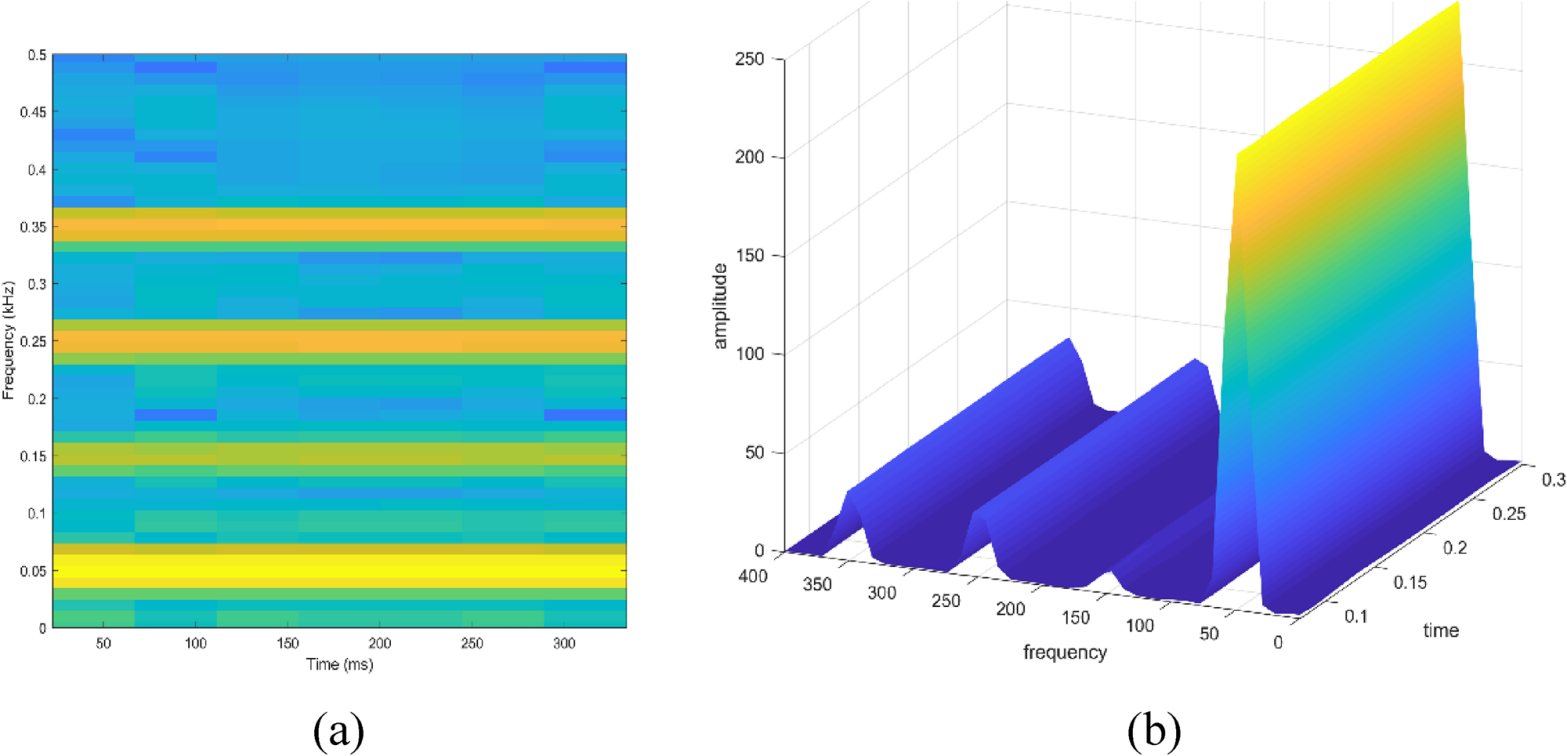
(**a**) Spectrogram of harmonics signal (**b**) STFT spectrum.

**Figure 21:**
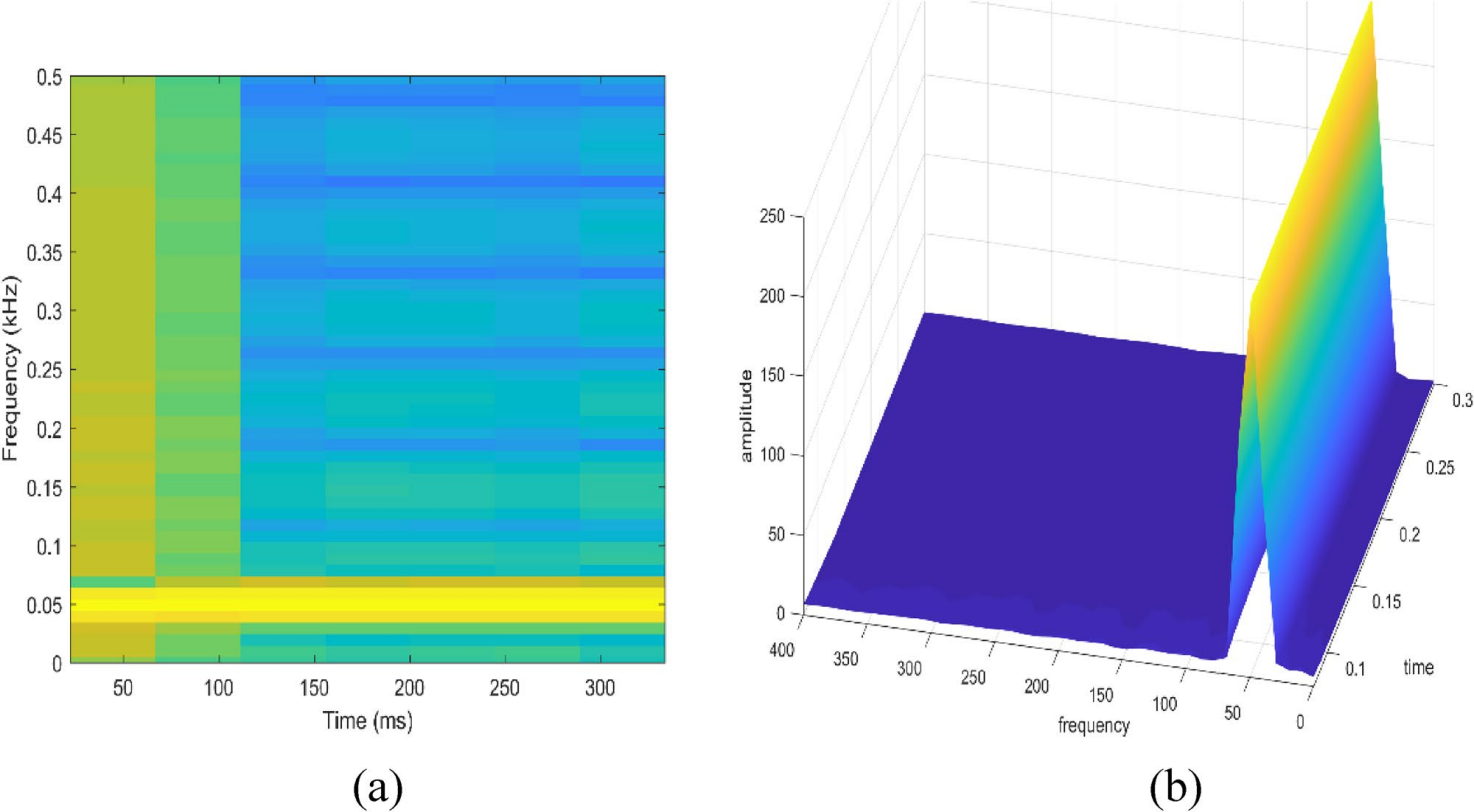
(**a**) Spectrogram of transient signal (**b**) STFT spectrum.

**Figure 22:**
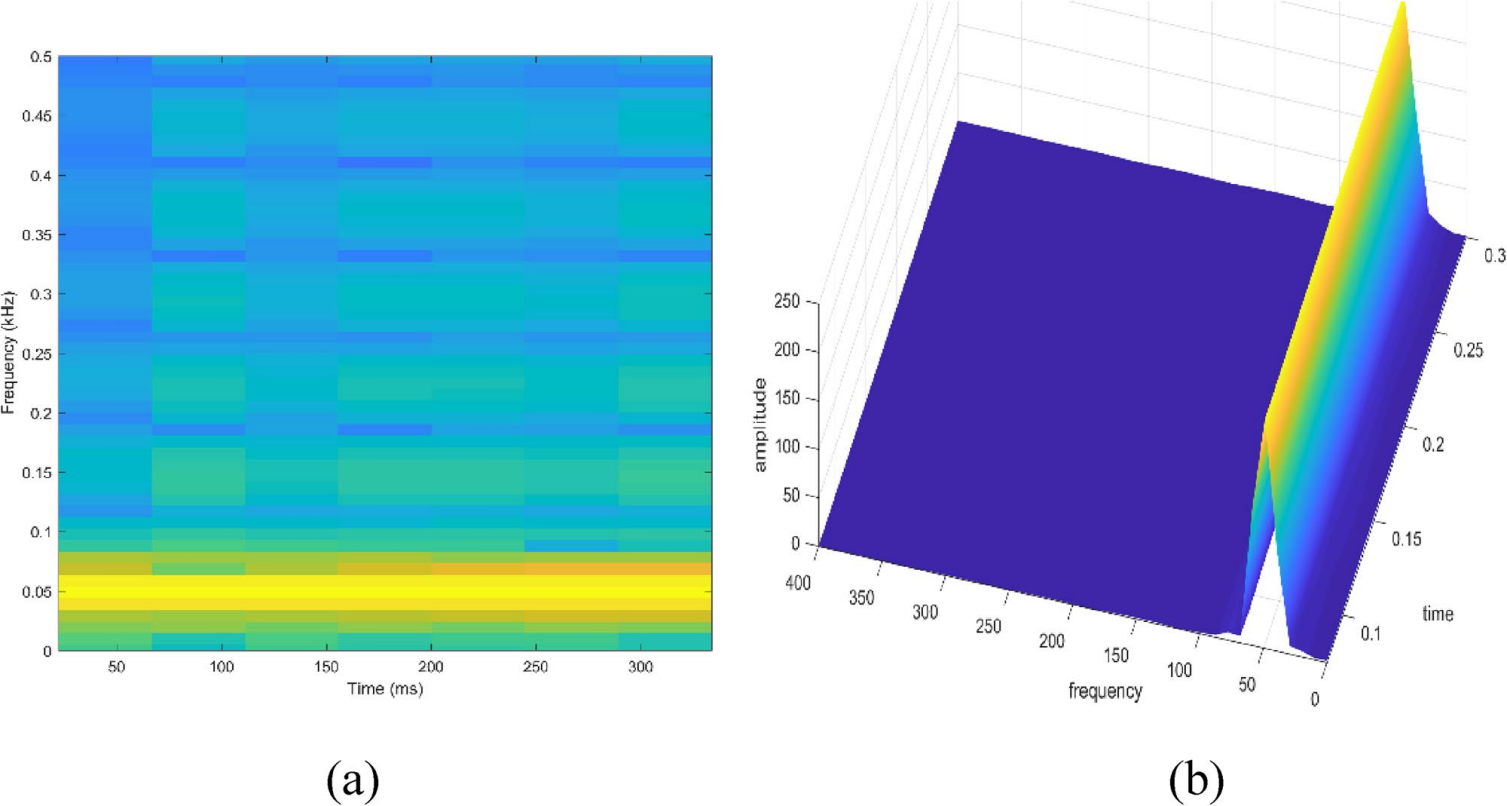
(**a**) Spectrogram of fluctuations signal (**b**) STFT spectrum.

**Figure 23:**
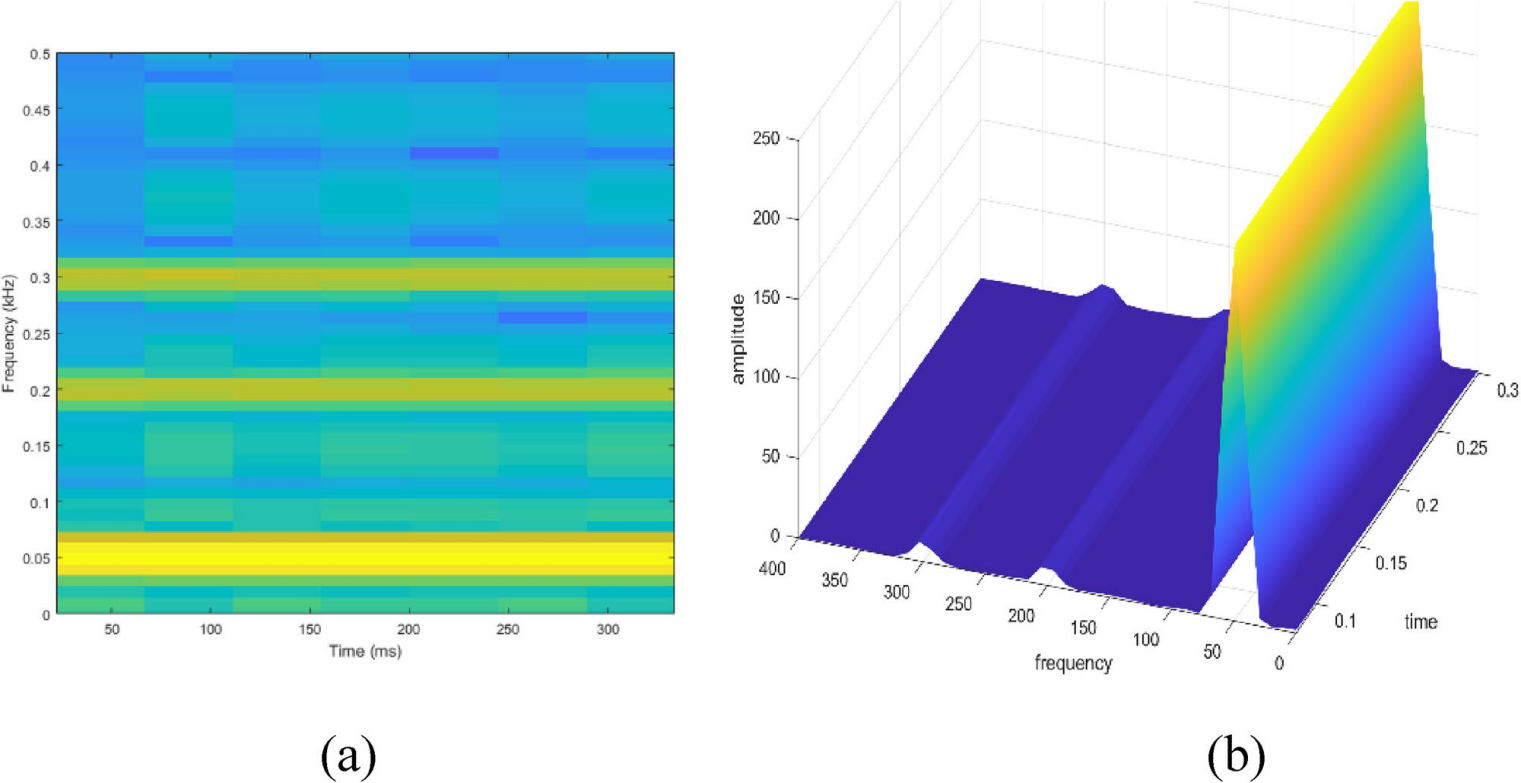
(**a**) Spectrogram of flicker signal (**b**) STFT spectrum.

**Figure 24:**
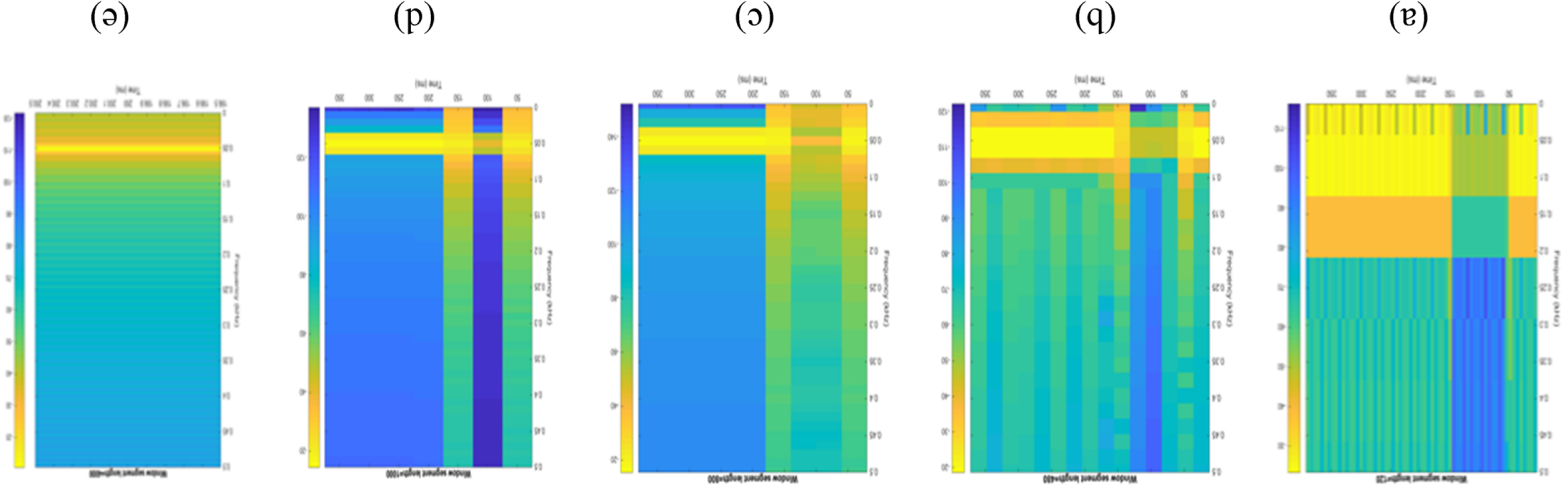
Interruption signal analyzed using STFT for different window sizes of segment length: (**a**) 120 (**b**) 480 (**c**) 800 (**d**) 1000 (**e**) 4000.

Signal to be transformed, window function and time index are represented by $x\left(t\right), \omega \left(\tau \right)$ and $\tau$. $X(\tau ,\omega )$ is the Fourier transform of $x\left(t\right)\omega (t-\tau )$ and represents a complex function. The complex values represent phase and magnitude of signal over time and frequency. ‘Hamming window’ is used for analysis of PQ signals. Figures 16, 17, 18, 19, 20, 21, 22, 23 and 24 show the analysis of all the PQ disturbance signals using STFT. The spectrogram is considered as STFT representation of the signals and STFT spectrum in all the figures represent a two-dimensional representation of frequency and time with varying amplitude indicated by common color bar in Fig. 16.

To depict the change in signal representation for changing window size, an interruption signal is considered as an example. Figure 24 shows the variation of an interruption signal, for different widths of window segments. 

Precise window size is necessary for STFT i.e. window size must neither be too small nor too large such that information is lost in representation. Blue segments show low power levels and broad yellow color in the spectrogram shows signal power spread across the range of frequencies. As window size is increased, good frequency resolution is possible by loosing time information and as window size is narrowed down, good time resolution is possible by loosing frequency information. Selection of window size is necessary for balancing both resolutions. By using STFT, frequency versus time information can be obtained by proper choice of window width. The perception of signal changes upon changing the window size. This has initiated for wavelet transform based methods.

## Application of continuous wavelet transform (CWT)

The signals which are a function of time can be transformed into another domain of time and frequency for better interpretation of the original signal in time domain. Continuous wavelet transform, a mathematical transform technique, is used for analysis of signals. Detection of changes in signals using continuous wavelet transform is termed as continuous wavelet analysis in MATLAB. As voltage signal is a function of only time, this analysis is one-dimensional (1D) analysis. 

### Continuous wavelet analysis

**Figure 25:**
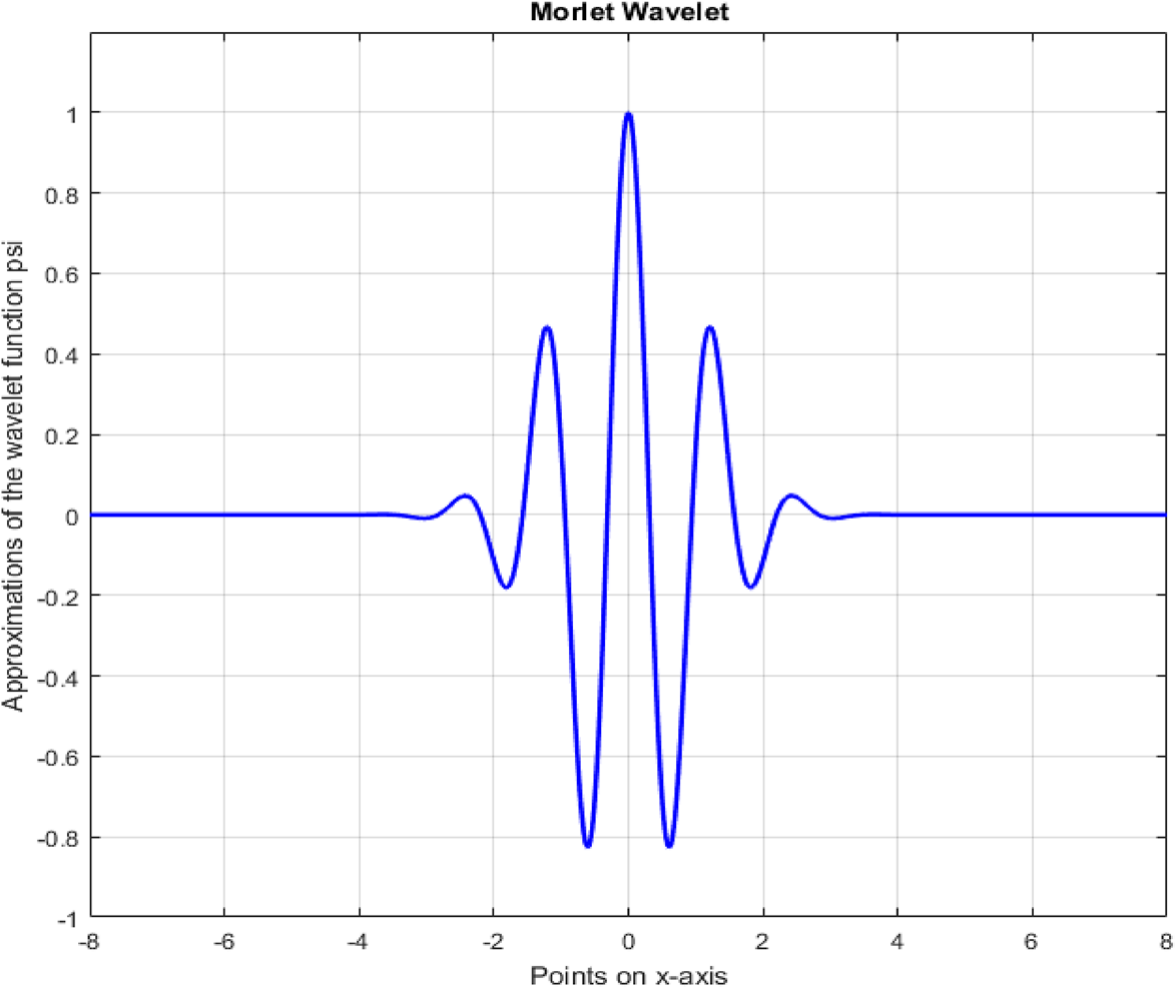
Morlet Wavelet.

A function in time domain is mapped into function of time and frequency by using continuous wavelet transform. A wavelet is chosen as mother wavelet indicated by $\psi$. For applying continuous wavelet transform (CWT), terms $s$ and $\tau$ are used. The term $s$ is scaling (stretching if $\left|s\right|>1$ or compressing if $\left|s\right|<1$) or dilation factor to control width of wavelet and the term $\tau$ is translation (shifting position in time) parameter to control location of the wavelet with $s, \tau \in R$ and $s>0$. Each wavelet is created by scaling and translation of mother wavelet which is a function that oscillates with finite energy and has zero mean value^26^. Different wavelet families are Haar, Daubechies, biorthogonal, Coiflets, Symlets, Morlet, Mexican Hat and Meyer wavelets. Morlet wavelet, shown in Fig. 25, is taken as mother wavelet as for majority of applications of CWT uses Morlet wavelet. Morlet wavelet has no scaling function, and has only wavelet function $\psi$, but is explicit^27^.

Scaling and translating a mother wavelet $\psi$ give a family ${\psi }_{\tau ,s}$ of ‘wavelet daughters’ given by Eq. (13)^28^. The correlation between the voltage variation signals and ‘wavelet daughters’ also termed as template functions, gives information about the disturbance in the signal. This is due to the comparison of template functions against the voltage variation signals.

13 $${\psi }_{\tau ,s}\left(t\right)=\frac{1}{\sqrt{s}}\psi \left(\frac{t-\tau }{s}\right)$$

CWT with respect to the wavelet $\psi$ is ${W}_{x,\psi }\left(\tau ,s\right)$, given by Eq. (14) is the wavelet transform by mapping the signal in time domain $x(t)$ into a function of $s$ and $\tau$ giving information simultaneously on time and frequency, where scale is related inversely to frequency^28^. Position of wavelet in time domain is given by $\tau$ and position in frequency domain is given by $s$.

14 $${W}_{\tau ,s}\left(x(t)\right)=\langle x\left(t\right),{\psi }_{\tau ,s}\left(t\right)\rangle ={\int }_{-\infty }^{\infty }x\left(t\right){\psi }_{\tau ,s}^{*}(t)dt=\frac{1}{\sqrt{s}}{\int }_{-\infty }^{\infty }x(t){\psi }^{*}\left(\frac{t-\tau }{s}\right)dt$$

It can be established that by using CWT, time domain functions can be mapped into time and frequency domain. Sum over all time of the signal multiplied by scaled, shifted versions of the wavelet function $\psi$ defines CWT and coefficients as function of scale and position are obtained^27^.

### Continuous wavelet analysis using MATLAB graphical interface

**Figure 26:**
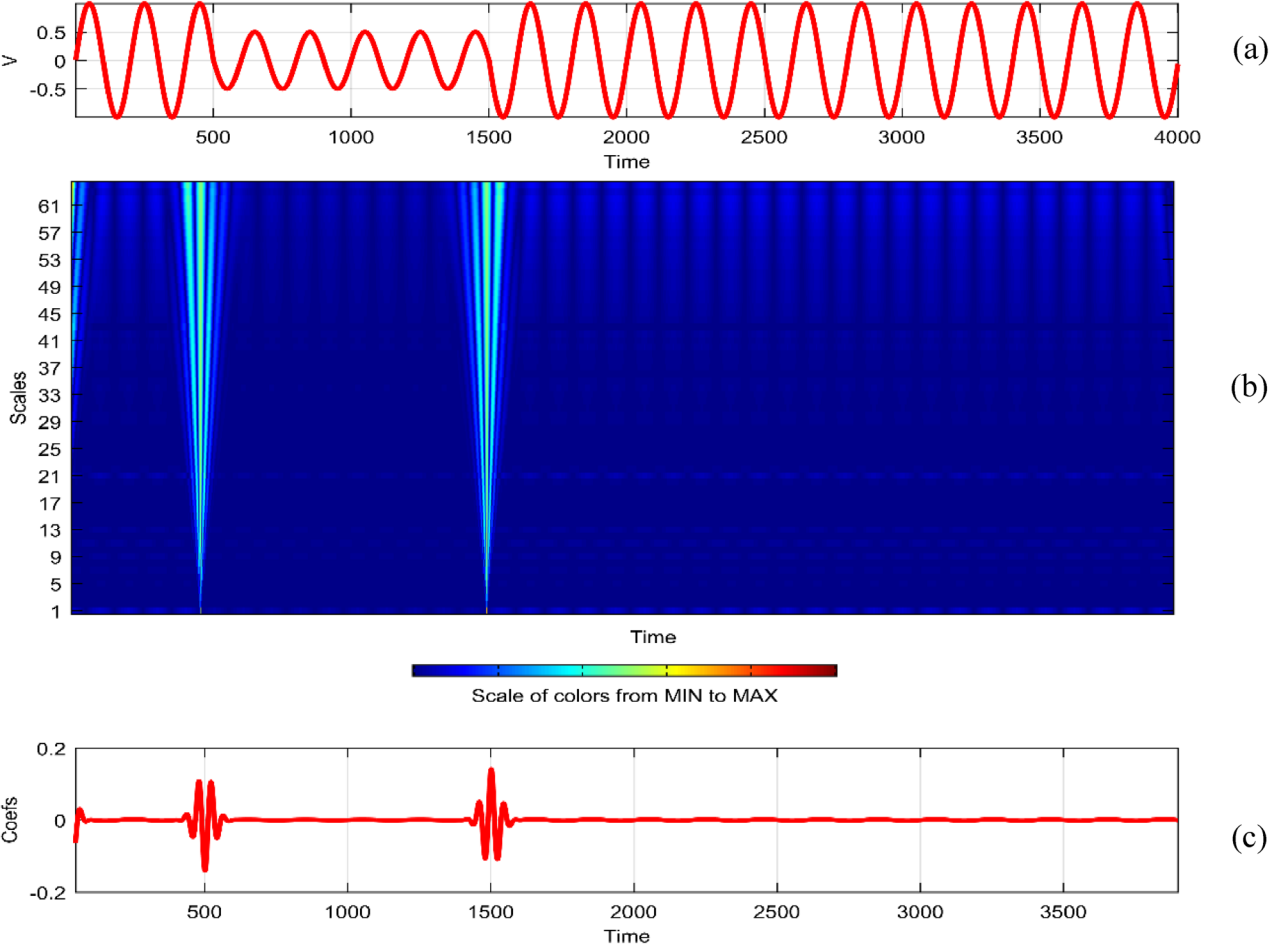
(**a**) Sag (**b**) Absolute CWT Coefficients (**c**) Coefficients line.

**Figure 27:**
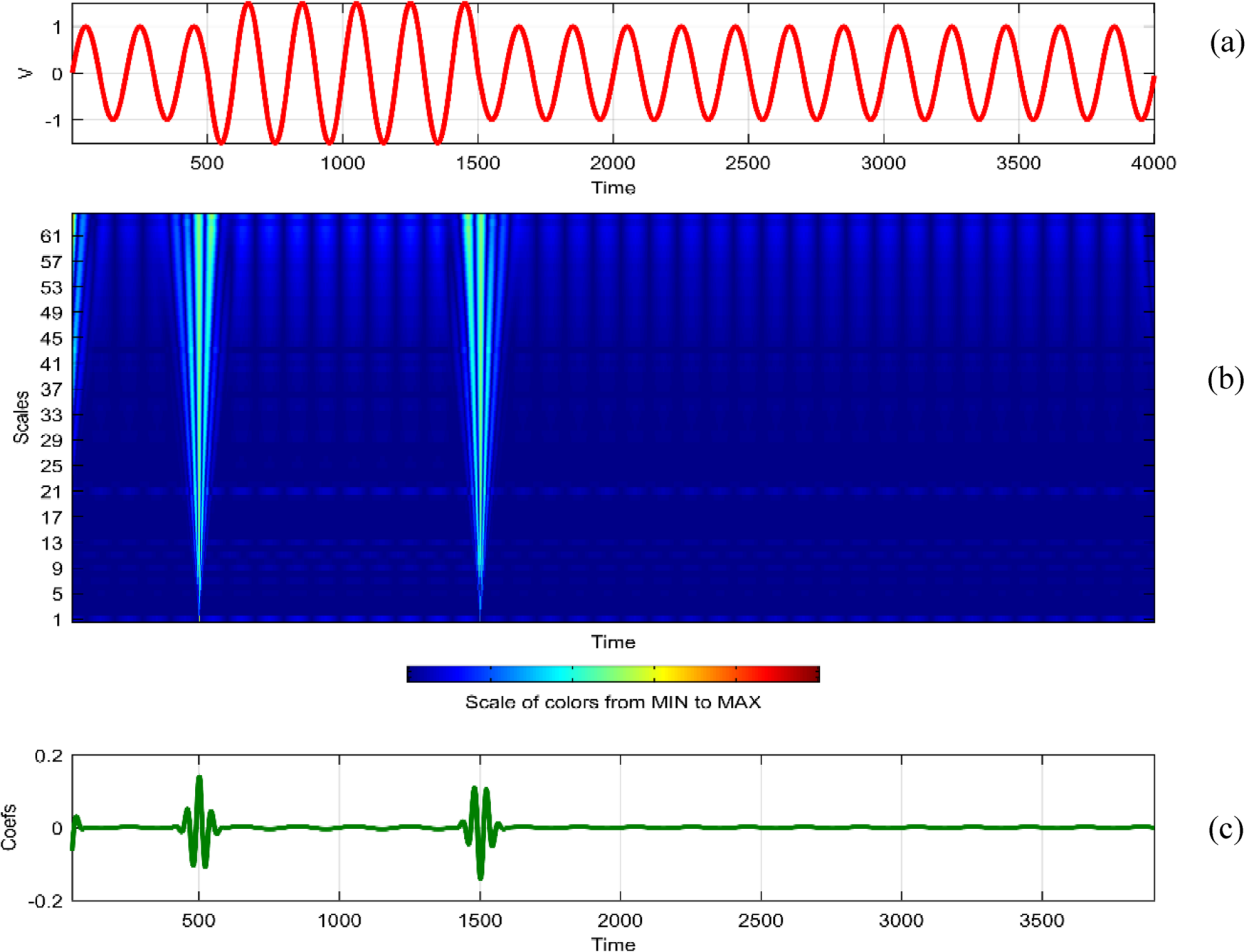
(**a**) Swell (**b**) Absolute CWT Coefficients (**c**) Coefficients line.

**Figure 28:**
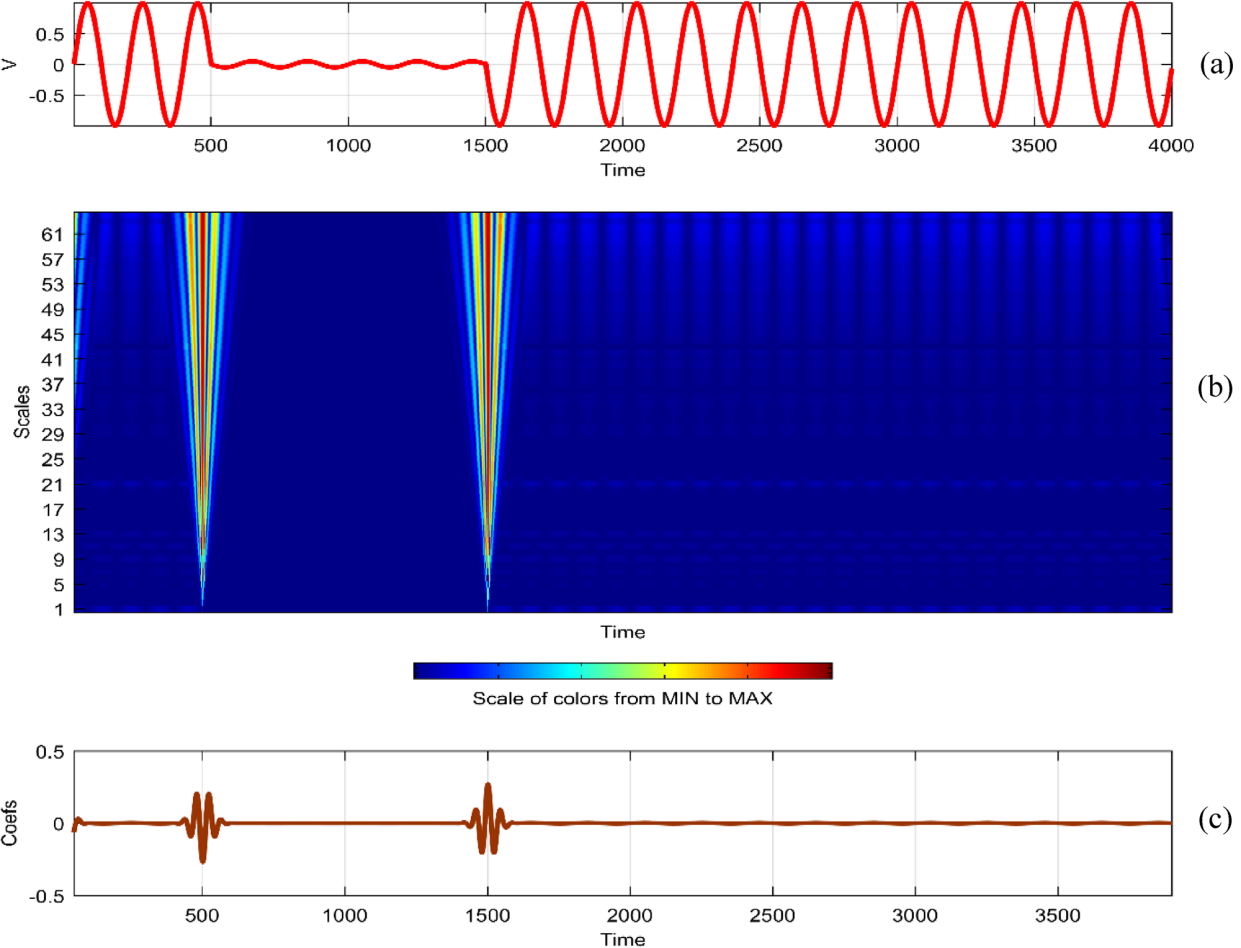
(**a**) Interruption (**b**) Absolute CWT Coefficients (**c**) Coefficients line.

**Figure 29:**
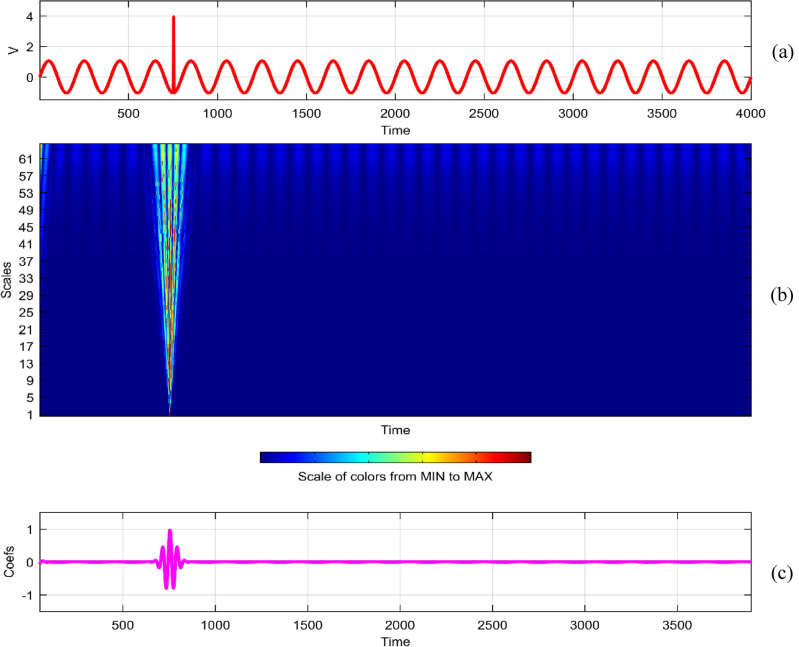
(**a**) Transient (**b**) Absolute CWT Coefficients (**c**) Coefficients line.

**Figure 30:**
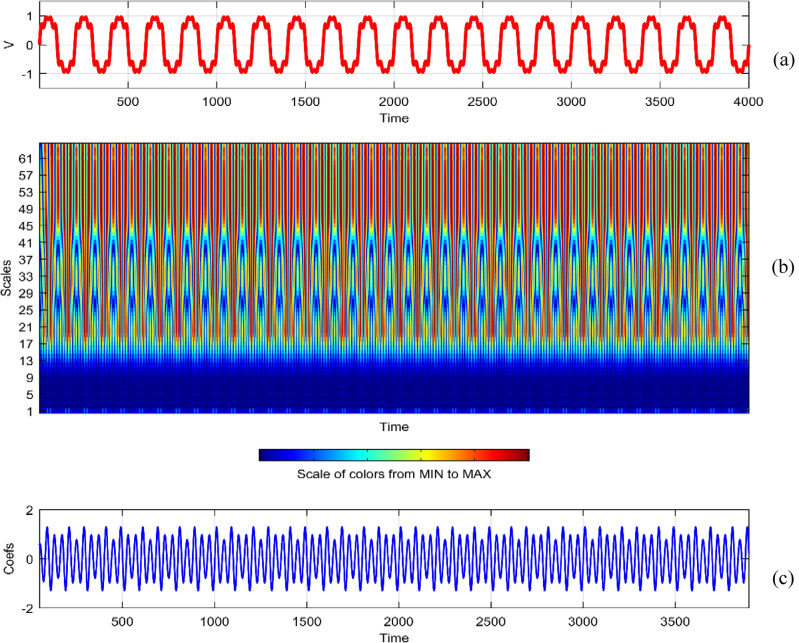
(**a**) Harmonics (**b**) Absolute Coefficients of CWT (**c**) Coefficients line.

**Figure 31:**
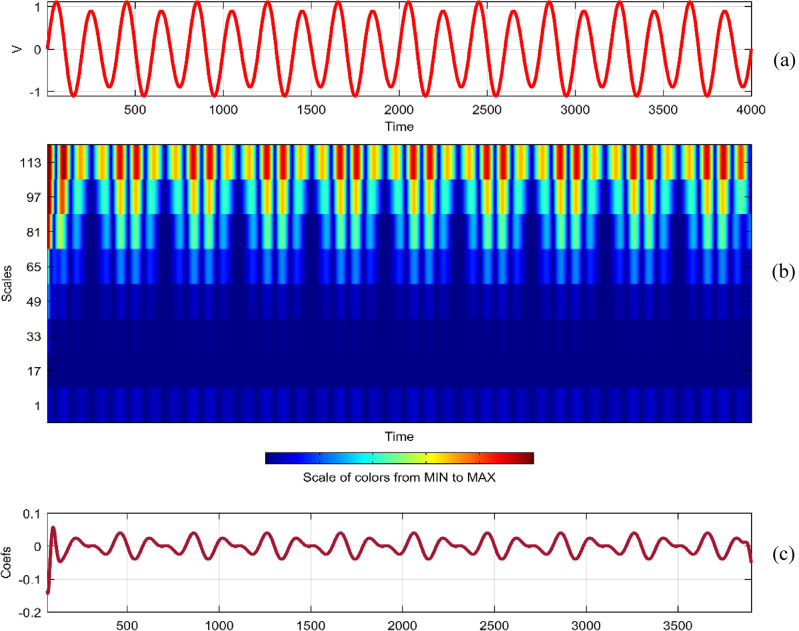
(**a**) Fluctuations (**b**) Absolute CWT Coefficients (**c**) Coefficients line.

**Figure 32:**
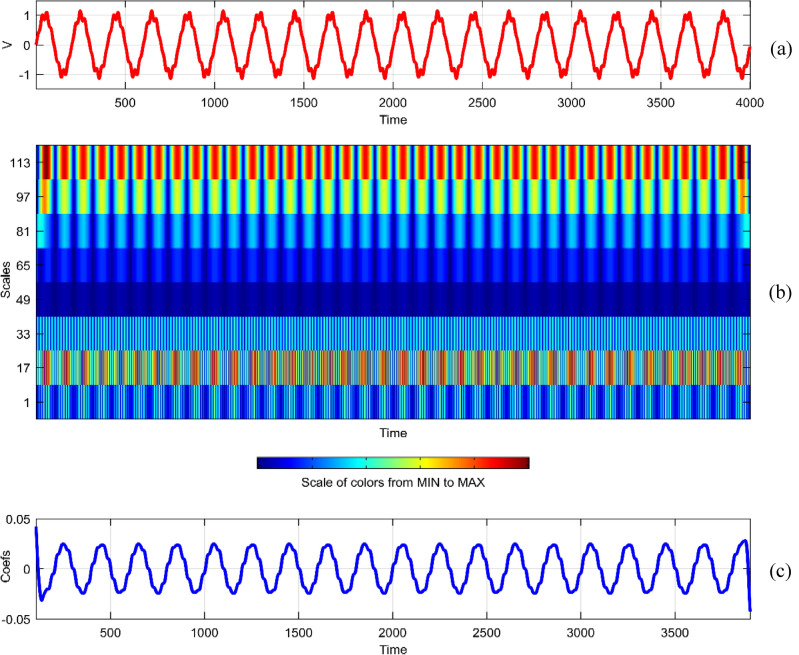
(**a**) Flicker (**b**) Absolute CWT Coefficients (**c**) Coefficients line.

Continuous Wavelet Analysis (CWT) is performed using MATLAB, leveraging both command-line functionality and the graphical interface for a comprehensive exploration of power quality disturbance signals^29,30^. MATLAB's graphical interface provides an intuitive platform for users to interactively analyze and visualize CWT results. The combination of command-line scripts and graphical tools enhances the accessibility and user-friendliness of the analysis process. MATLAB's graphical interface facilitates the dynamic exploration of CWT results by allowing users to interactively adjust scale and time parameters^31^. This interactivity empowers researchers to fine-tune the analysis, enabling a detailed examination of disturbances at different scales and time intervals^32^. The graphical representation of CWT coefficients as a function of scale and time offers a unique perspective on signal variations. MATLAB's plotting capabilities enable the creation of coefficient line plots, aiding in the identification of hidden patterns that might not be immediately apparent in the original signals. MATLAB's graphical interface provides a wide selection of wavelets for CWT analysis. Researchers can easily experiment with various mother wavelets, including Morse, Morlet, and bump wavelets, to identify the most suitable wavelet for capturing specific features in power quality disturbances^33,34^. The inclusion of 3D plots representing disturbance signals with time, scale, and coefficient values enhances the visual interpretation of CWT results. These plots, generated through MATLAB's graphical interface, offer a holistic view of energy distribution across different scales and times^35,36^. Researchers can utilize MATLAB's graphical interface to obtain quantitative insights into the energy levels of CWT coefficients. The tabular representation of energy values for different scales (1 and 64) aids in expressing signal strength in terms of coefficient energy^37^. The integration of MATLAB's graphical interface into the CWT analysis process not only simplifies the workflow but also contributes to the enhanced interpretability of power quality disturbance signals. Wavelet toolbox is one of powerful graphical interfacing tools in MATLAB for power quality^38^. In^39^, sag and swell are analyzed in a transmission system employing a suitable compensator. Harmonics are analyzed using STFT in^40^ using different window lengths and in^41^, statistical features are extracted from PQ signals. Wavelet toolbox main menu can be opened in a new window and continuous Wavelet 1-D graphical tool is selected. The signal can be loaded directly in “**.** mat” format or MAT-files which refers to files readable by MATLAB. The signal can also be imported from workspace in MATLAB. Similar results can be obtained in both ways. Signals considered are transient, sag, swell, interruption, harmonics, fluctuations and flicker for duration of 0.4 s. The coefficients plot as a function of scale and time and coefficients line are obtained in step-by-step mode and the coefficients line is a plot of the coefficients of 64-by-4001 size matrix. The coefficients line plot also gives information about the changes in the signal. It is observed from coefficients line plot, during disturbances, a deviation is observed in the coefficients. Mother wavelet is selected as “morl”, indicating Morlet wavelet. By clicking on the “Analyze” button continuous wavelet transform is performed. The maximum values of coefficients lines are calculated for scale of 64. Scale value of 32 has frequency 0.025. It is observed from all the plots that continuous wavelet analysis using continuous wavelet transform provides same coefficients using either one of command line interface or wavelet toolbox. The additional feature included in toolbox is selected axes can be any one of, two of or all three of coefficients, coefficients line, and maxima lines. In Figs. 26, 27, 28, 29, 30, 31 and 32, the original disturbance signals are shown along with coefficients and coefficients line for scale value of 32.

For scale value of 64, CWT coefficients of sag signal have a maximum value of 0.4996 at 0.1501 s.

For scale value of 64, CWT coefficients of swell signal have a maximum value of 0.5117 at 0.1458 s.

For scale value of 64, CWT coefficients of interruption signal have a maximum value of 0.9452 at 0.1501 s.

For scale value of 64, CWT coefficients of transient signal have a maximum value of 0.5609 at 0.0753 s.

For scale value of 64, CWT coefficients of harmonics signal have a maximum value of 0.9469.

For scale value of 64, CWT coefficients of fluctuations signal have a maximum value of 0.2062.

In the context of continuous wavelet analysis, the evaluation of the flicker signal at a scale value of 64 reveals its maximum CWT coefficient, reaching a pivotal value of 0.1140. This peak coefficient serves as a critical indicator for discerning variations initiated in the voltage, offering insights into the dynamic changes within the signal and facilitating the visualization of deviations from the ideal waveform^42^. The choice of Morse, Morlet, and bump wavelets as mother wavelets adds depth to the analysis, each contributing unique characteristics to the exploration of power quality disturbance signals. These wavelets play a pivotal role in uncovering hidden patterns that might elude detection in the original signals^43,44^. The matrix dimensions of coefficients for all considered signals are contingent on the selected scale, with the scale range of 1:16:128 dictating the breadth of frequency representations. This comprehensive scaling approach allows for a detailed exploration of signals across a spectrum of frequencies, providing a foundation for nuanced analyses. Tables 1 and 2 further enrich the narrative; Table 1 meticulously delineates the energy values of coefficients obtained through the continuous wavelet transform at scales 1 and 64, offering a dual-scale perspective on high and low frequencies. Meanwhile, Table 2 introduces a three-dimensional (3D) visualization paradigm, encapsulating disturbance signals with time, scale, and coefficient values on the respective axes. This visual representation at scales 1 and 64 unravels the intricate interplay between high and low-frequency components, emphasizing the inverse relationship between scale and frequency.

**Table 1:** Energy values of CWT coefficients for each disturbance.

Signal	Scale 1	Scale 64
Sine	0.0015	76.327
Sag	0.0001	96.386
Swell	0.0002	109.39
Interruption	0.0001	159.86
Harmonics	0.0087	206.68
Transient	0.0339	340.59
Flicker	0.0006	76.485
Fluctuations	0.0001	82.773

**Table 2:** Continuous wavelet transform based 3D plots of coefficients.

Colormap used for indicating magnitude of coefficients	
Signal	3D plot
Sag	
Swell	
Interruption	
Transient	
Harmonics	
Fluctuations	
Flicker	

The examination of power quality disturbance signals during their initiation and recovery phases reveals conspicuous deviations in the coefficients, providing crucial insights into the transient nature of these disturbances^45,46^. To unravel the intricacies of these signals, a comprehensive analysis employing Fourier transform, short-time Fourier transform, and continuous wavelet transform (CWT) is undertaken. Each of these transforms serves as a lens through which the signals are perceived in different domains, enriching the understanding of their multifaceted characteristics^47,48^. Notably, CWT is applied with continuous scales, signifying an inverse relationship with frequency^49^. This approach allows for the encapsulation of diverse energy levels within each scale, with the highest scale value of 64 strategically employed to extract low-frequency contents, while the lowest scale value of 1 adeptly captures high-frequency components. Consequently, the information about power quality disturbances is encapsulated as a dynamic interplay between low and high-frequency energy levels. The coefficients derived from CWT are meticulously plotted as a function of both scale and time, elucidating the temporal and frequency-specific variations. This methodology proves instrumental in unveiling hidden patterns that remain obscured in the original signals^50,51^. However, it is imperative to acknowledge that the computational demands of CWT analysis introduce redundancy, necessitating judicious considerations^52^. The analytical process is facilitated through both command-line functionality and the graphical interface of MATLAB, providing a robust platform for the identification of power quality disturbances using the insights derived from CWT^53,54^.

## Discrete wavelet transform (DWT)

Discrete Wavelet Transform (DWT) emerges as a powerful tool for disentangling the intricate details within power quality disturbance signals^55,56^. Leveraging the concept of multiresolution analysis, DWT efficiently decomposes a signal into multi-resolution components, unraveling its diverse frequency components. Discrete wavelet transform is used for decomposing a signal into multi-resolution components and for detecting changes in signal waveforms^57^. The theory of multiresolution signal decomposition was proposed by Stephan Mallat and certain^58^ important theorems were proved with description of mathematical modes in which are necessary for multiresolution representation termed as, “*wavelet representation*” for extracting information between successive resolutions. The decomposition process unfolds in a hierarchical fashion, initially splitting the signal into level one approximation and detail^59^. The iterative refinement continues, progressively delving deeper into the signal's nuances, until a sufficient level of information is captured^60^. The elegance of multiresolution analysis unfolds as the signal undergoes a sequential dissection, revealing its nuanced structure^61^. Initially, the signal is bifurcated into a level one approximation and detail. An iterative refinement process ensues, as the detail is disregarded, and the approximation is further scrutinized through the lens of a secondary multiresolution analysis^62^. This cascading refinement continues until a critical juncture is reached where the loss of information becomes perceptible. The quintessence of wavelet analysis lies in the identification of signal variations through this intricate multiresolution journey. The discrete wavelet transform (DWT) becomes the linchpin, orchestrating this process with finesse. It orchestrates a dual representation of the signal—low frequency encapsulated within the approximation and high frequency articulated through detail components^63,64^. In stark contrast to its continuous counterpart, DWT operates with optimal efficiency, eliminating redundancy while preserving the essential information mosaic. The overarching aim remains clear: applying the wavelet transform as a discerning lens to unravel the mysteries of power quality disturbances, decoding the nuanced features embedded within the signals^65^. This pursuit is augmented by the extraction of key attributes from the level 1 detail coefficients, encompassing the peak characteristics, variance, and skewness of level 7 approximations, alongside the mean deviation of level 6 details, as explicated in^66^.

**Figure 33:**
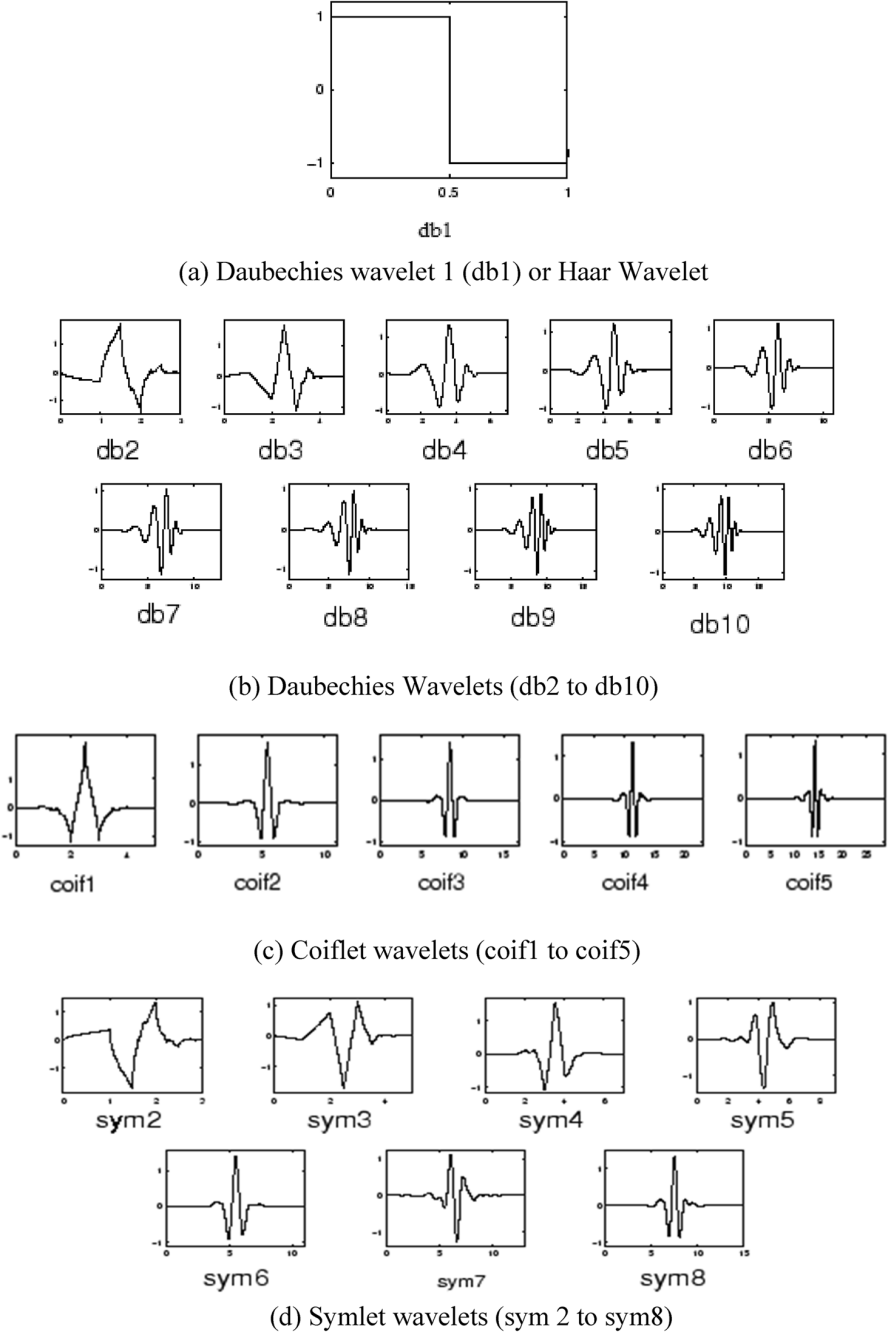
Different Wavelet families.

It is mentioned in^67^ that the property of multiresolution gives precise low and high frequency content information of the analyzed signal by using long and short windows. Figure 33 comprises of few wavelets (a) db1 (b) db2 to db10 (c) coif 1 to coif 5 (d) sym 2 to sym8 which are used as mother wavelets. The discrete wavelet transform is defined in Eq. (15)^6^, with complex conjugate of mother wavelet given by ${\psi }^{*}(t)$.

15 $${{\text{DWT}}}_{\uppsi }\left({\text{m}},{\text{n}}\right)=\underset{-\infty }{\overset{+\infty }{\int }}{\text{x}}\left({\text{t}}\right){\uppsi }_{{\text{m}},{\text{n}}}^{*}\left({\text{t}}\right){\text{dt}}$$

In Eq. (15), the discretized mother wavelet is given by Eq. (16)

16 $${\uppsi }_{{{\text{m}},{\text{n}}}} \left( {\text{t}} \right) = {\text{a}}_{0}^{{ - {\text{m}}/2}} {\uppsi }\left( {\left( {{\text{t}} - {\text{na}}_{0}^{{\text{m}}} {\text{b}}_{0} } \right)/{\text{a}}_{0}^{{\text{m}}} } \right)$$

In DWT, scaling and translation parameters $a$, $b$ are discretized as $a={a}_{0}^{m}$ and $b=n{b}_{0}{a}_{0}^{m}$, where ${a}_{0}>1$,  ${b}_{0}>0$, and $m$, $n$ are positive integers. Dilation refers to scaling i.e. change of frequency. Translation refers to shifting of mother wavelet^68^. Any function in time domain is represented by discrete wavelet transform with scaling function as $\varphi \left(t\right)$ and wavelet function as $\psi \left(t\right)$^6^ is represented by Eq. (17).

17 $${\text{f}}\left({\text{t}}\right)=\sum_{{\text{k}}}{{\text{c}}}_{{\text{j}}}({\text{k}}){2}^{{\text{j}}/2}\mathrm{ \varphi }\left({2}^{{\text{j}}}{\text{t}}-{\text{k}}\right)+ \sum_{{\text{k}}}{{\text{d}}}_{{\text{j}}}({\text{k}}){2}^{{\text{j}}/2}\uppsi \left({2}^{{\text{j}}}{\text{t}}-{\text{k}}\right)$$

The term $\sum_{{\text{k}}}{{\text{c}}}_{{\text{j}}}({\text{k}}){2}^{{\text{j}}/2}\mathrm{ \varphi }\left({2}^{{\text{j}}}{\text{t}}-{\text{k}}\right)$ represents approximation and $\sum_{{\text{k}}}{{\text{d}}}_{{\text{j}}}({\text{k}}){2}^{{\text{j}}/2}\uppsi \left({2}^{{\text{j}}}{\text{t}}-{\text{k}}\right)$ represents detail of the signal with ${\text{j}}$ referring ‘scaling parameter’ and ${\text{k}}$ ‘shift parameter’.

### DWT based Identification of PQ disturbances

Embarking on a meticulous dissection, power quality disturbance signals undergo a comprehensive analysis through a sophisticated five-level decomposition, leveraging the prowess of Daubechies 4 (db4) as the designated mother wavelet^69,70^. This intricate process involves the instantiation of daughter wavelets, aptly referred to as template functions, with their width defining the elusive scale. The multifaceted procedure unfolds through the tandem application of filtering and downsampling, the dynamic duo driving the dissection into quintessential components^71^. At the heart of this analytical symphony lies the extraction of linear combinations of wavelet functions, christened as wavelet coefficients^72^. This culmination not only marks the transformation of the signal but births a new realm—the wavelet transform^73^. The convolutional interplay with low and high pass filters ushers in a dichotomy of approximation and detail coefficients, each holding a unique key to the intricate tapestry of the signal's essence. To crystallize this revelation, the obtained signal undergoes a deliberate downsampling by a factor of two, culminating in a nuanced and distilled representation^74,75^.

**Figure 34:**
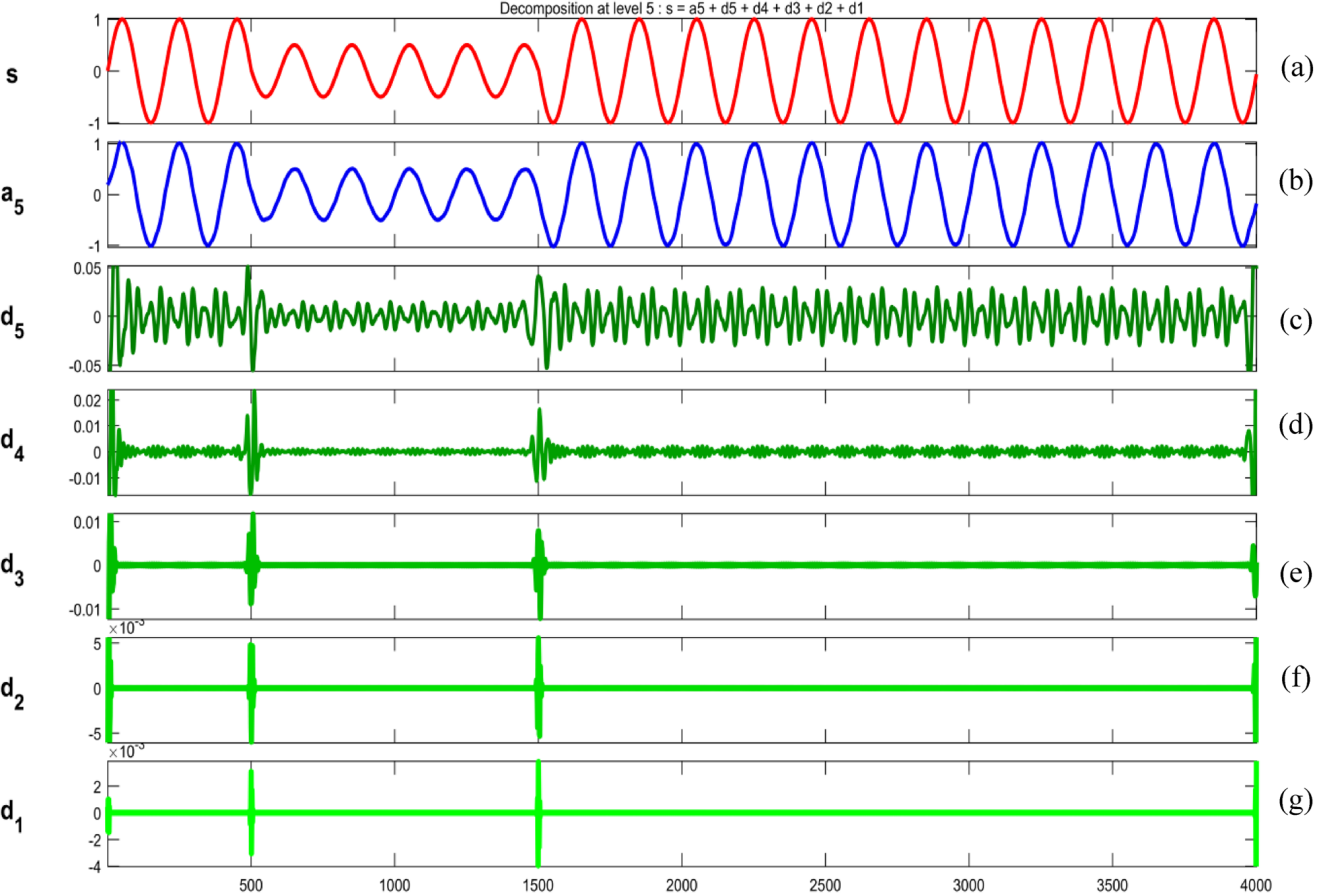
(**a**) Sag (**b**) Approximation (level 5) (**c**) to (**g**) 5th, 4th, 3rd, 2nd and 1st level details.

**Figure 35:**
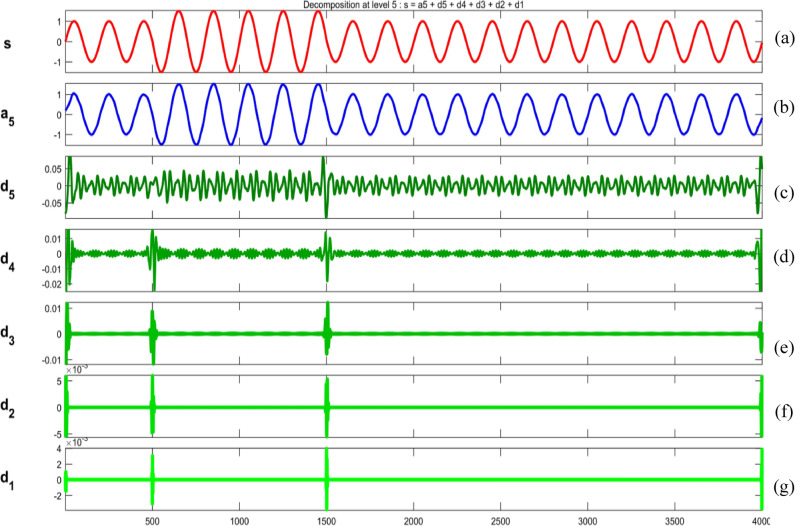
(**a**) Swell (**b**) Approximation (level 5) (**c**) to (**g**) 5th, 4th, 3rd, 2nd and 1st level details.

**Figure 36:**
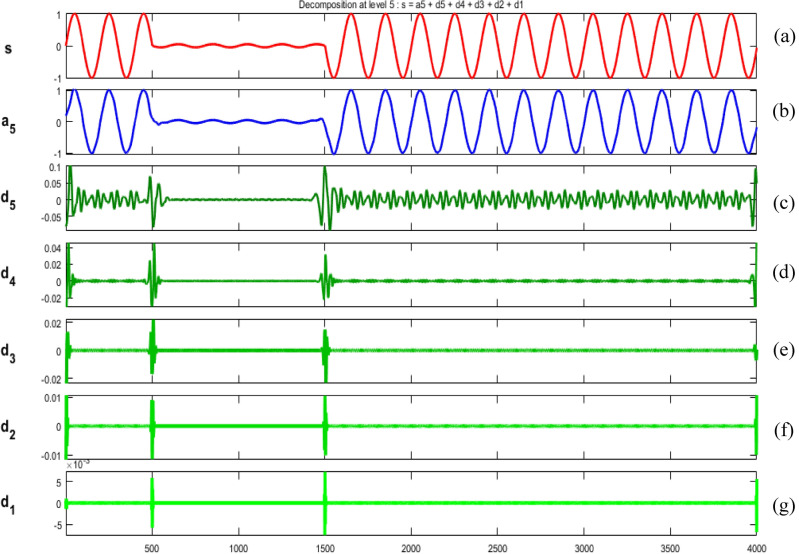
(**a**) Interruption (**b**) Approximation (level 5) (**c**) to (**g**) 5th, 4th, 3rd, 2nd and 1st level details.

**Figure 37:**
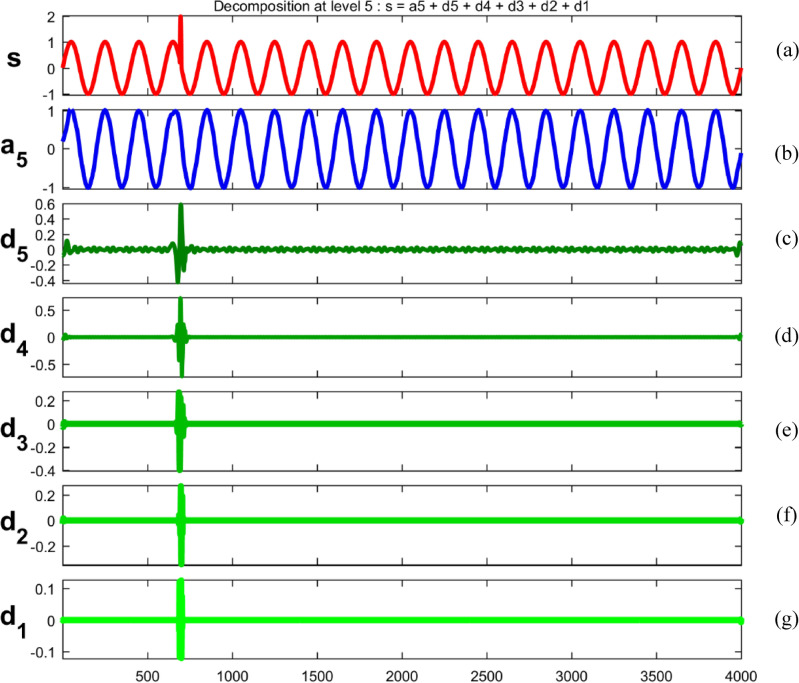
(**a**) Transient (**b**) Approximation (level 5) (**c**) to (**g**) 5th, 4th, 3rd, 2nd and 1st level details.

**Figure 38:**
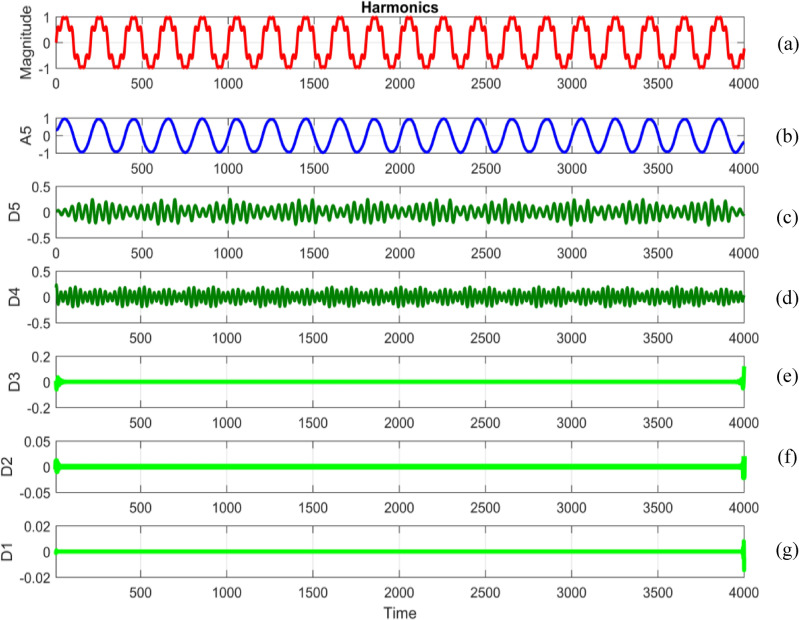
(**a**) Harmonics (**b**) Approximation (level 5) (**c**) to (**g**) 5th, 4th, 3rd, 2nd and 1st level details.

**Figure 39:**
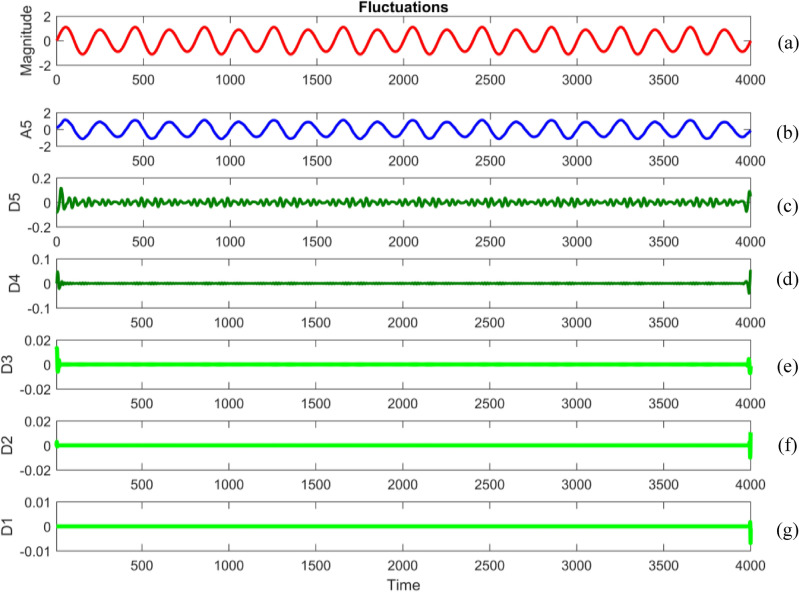
(**a**) Fluctuations (**b**) Approximation (level 5) (**c**) to (**g**) 5th, 4th, 3rd, 2nd and 1st level details.

**Figure 40:**
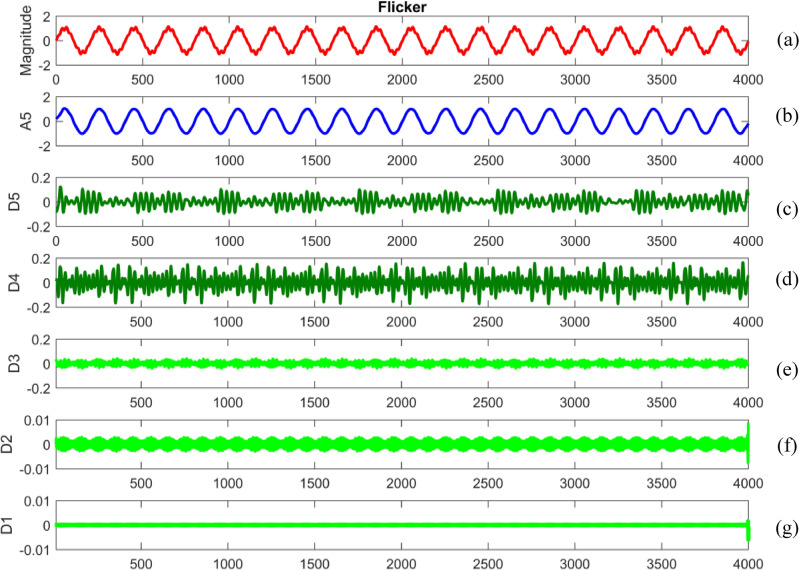
(**a**) Flicker (**b**) Approximation (level 5) (**c**) to (**g**) 5th, 4th, 3rd, 2nd and 1st level details.

Figures 34, 35, 36, 37, 38, 39 and 40 show extraction of level five approximations and level five to one details from signals. From 500th sample and to 1500th sample out of 4000 samples of time disturbances of sag, swell and interruption are initiated. Following information is obtained from few of the decomposed signals:

- The maximum values of details for levels one to five of transient are 0.9970, 0.4357, 0.8022, 0.4972 and 0.2626.
- In harmonics, fluctuations and flicker signals, there are no abrupt changes but a change in frequency of signals can be observed. So initiation and recovery of disturbances cannot be directly perceivable from the signals.
- The maximum values of details for levels one to five of harmonics are 0.0108, 0.0321, 0.1286, 0.2385 and 0.2511.
- When ‘db20’, ‘coif5’ and ‘db37’ wavelets are used, details for all levels are zero. If ‘db4’ wavelet is used, the maximum values of details for levels one to five of fluctuations are 0.0056, 0.0158, 0.0134, 0.0518 and 0.1171.
- The maximum values of details for levels one to five of flicker are 0.0051, 0.0165, 0.0402, 0.1645 and 0.1242.

**Figure 41:**
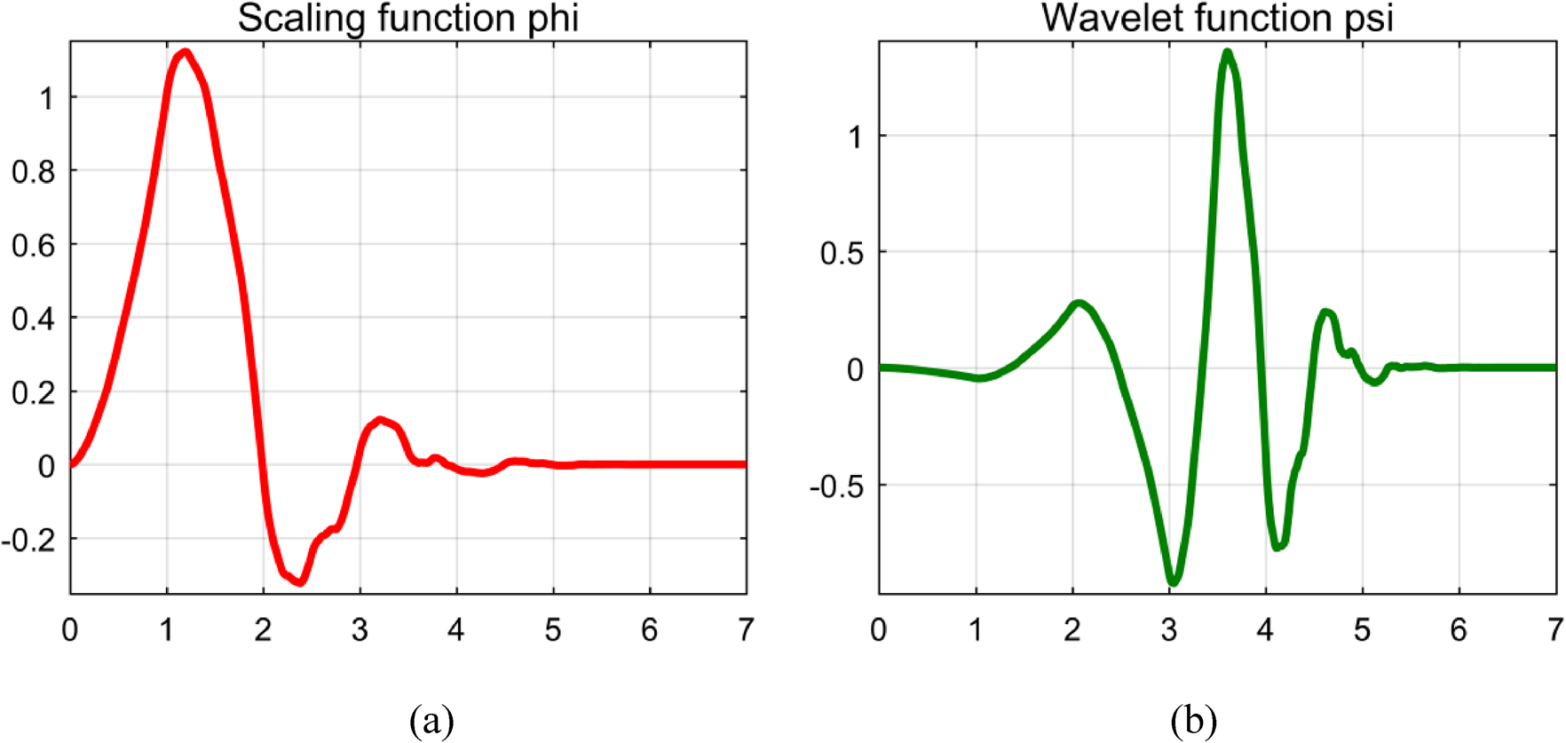
Daubechies (‘db4’) wavelet (**a**) Scaling function (**b**) Wavelet function.

Navigating the intricate landscape of signal analysis, the Discrete Wavelet Transform (DWT) emerges as a beacon of precision, adept at discerning nuanced transitions within the signal. This methodical dissection unfolds over five levels, where the signal undergoes a metamorphosis, decomposing into a tapestry of multi-resolution components. The linchpin of this process lies in the intricate interplay of template functions—a consequence of the shifting and dilation of the chosen mother wavelet. These templates, resembling a symphony of patterns, are meticulously compared with the disturbances encoded within the signal. The extent of correlation serves as a compass, revealing the distinct fingerprint of each type of disturbance. Daubechies fourth-order wavelet (db4), showcased in the visual tableau of Fig. 41, emerges as the maestro in power quality analysis. Its selection is not arbitrary but rooted in the 'compactness and localization' properties, rendering it an ideal candidate for unraveling the complexities of disturbances^76^. A pivotal study^77^ adds an extra layer of validation, drawing comparisons between the entropy values of approximations within disturbance signals and a reference sinusoidal signal—a testament to the robustness and reliability of the chosen methodology.

In the realm of signal analysis, the Discrete Wavelet Transform (DWT) stands out as a meticulous tool capable of discerning intricate transitions within signals^78^. Its remarkable ability to identify discontinuities is most pronounced at the initial level of detail. This intrinsic characteristic renders the DWT particularly adept at effectively identifying signals characterized by abrupt changes, overshadowing its efficacy in scenarios involving harmonics, fluctuations, and flicker, where alterations manifest more gradually. The lucidity of identification becomes notably conspicuous when scrutinizing signals associated with sag, swell, interruption, and transient phenomena^79^. Level 1 details, akin to a metaphorical magnifying lens, systematically unravel the complexities of these specific disturbances, furnishing a nuanced and exhaustive perspective crucial in the domain of power quality analysis^80^.

### Insights about the continuous wavelet transform (CWT) coefficient matrix and reason for selection of discrete wavelet transform (DWT) over CWT

- In the CWT coefficient matrix dimensions, each row of the matrix contains the CWT coefficients for one scale.
- The column dimension of the matrix is equal to the length of the input signal.
- There are 64 rows because the ‘SCALES’ input to CWT is 1:1:64, with 1 representing initial value, 1 representing increment and 64 representing maximum value of scale used.
- The length of different signals used in time domain is 2501 with input given in MATLAB as t = [0:0.0001:0.25].
- Thus, the CWT coefficient matrix has 64 rows and 2501 columns.
- Computation is more using continuous wavelet transform (CWT) and results in redundant information.
- Compared to CWT, discrete wavelet transform (DWT) contains required amount of information without redundancy and requires less computation.
- DWT is chosen over CWT as DWT results in a finite number of wavelet coefficients depending upon the integer number of the discretization step in scale and translation.
- DWT results in saving space**.**
- By using Multiresolution analysis in DWT, a signal can be decomposed into approximations (low frequency version) and details (high frequency version).
- The transitions present in the signal having abrupt changes can be easily captured from details by using DWT.

Power quality disturbances after applying Fourier, short-time Fourier, Continuous and discrete wavelet transforms are visualized in MATLAB.

## Conclusion

By applying different signal processing techniques to power quality disturbances, information about the signals can be extracted. Different signals are generated for a time duration and based on the type of disturbances, variations are shown in magnitude and frequency. Perception of power quality signals in different domains by applying mathematical transforms of Fourier transform, Short time Fourier transform, Continuous wavelet transform and Discrete wavelet transform is done. Results of application of Fourier Transform to power quality disturbances are signals in time domain are represented in frequency domain, existing frequency in the signals can be determined and the time at which frequency components exist cannot be determined. Fourier transform is effective for the signals whose frequency content is same at every point of time. Results of application of Short-Time Fourier Transform (STFT) to power quality disturbances are signals in time domain are represented in time and frequency domains and depends on window size. By using STFT, frequency versus time information can be obtained by proper choice of window width. Visual two-dimensional representation of the signal as a function of frequency and time can be obtained with varying magnitude. Results of application of Continuous Wavelet Transform (CWT) to power quality disturbances are by using CWT, coefficients as a function of scale and position can be obtained. Each scale stores different energy levels of the signal. Coefficients line plot gives information about changes in the signal. CWT results in better interpretation for finding hidden patterns that are not available in original signals. It is a time-scale analysis method. Results of application of Discrete Wavelet Transform (DWT) to power quality disturbances are signals can be decomposed into multiresolution components—approximations and details. Approximations are low frequency components. Details are high frequency components. Transition or singular points or any abrupt changes can be detected. It is a time-discretized scale analysis method. Fourier, Short Time Fourier, Continuous Wavelet transform and Discrete wavelet transforms are applied in MATLAB environment for power quality disturbances to provide perception in different domains giving useful information about the content that is not available in time domain signals.

## Data Availability

The datasets used and/or analyzed during the current study available from the corresponding author on reasonable request.
